# Disparities in COVID-19 Outcomes by Race, Ethnicity, and Socioeconomic Status: A Systematic-Review and Meta-analysis

**DOI:** 10.1001/jamanetworkopen.2021.34147

**Published:** 2021-11-11

**Authors:** Shruti Magesh, Daniel John, Wei Tse Li, Yuxiang Li, Aidan Mattingly-app, Sharad Jain, Eric Y. Chang, Weg M. Ongkeko

**Affiliations:** 1 Division of Otolaryngology–Head and Neck Surgery, Department of Surgery, University of California, San Diego; 2 Research Service, VA San Diego Healthcare System, San Diego, California; 3 The University of California Davis School of Medicine, Sacramento; 4 Department of Radiology, University of California, San Diego; 5 Radiology Service, VA San Diego Healthcare System, San Diego, California

## Abstract

**Importance:**

COVID-19 has disproportionately affected racial and ethnic minority groups, and race and ethnicity have been associated with disease severity. However, the association of socioeconomic determinants with racial disparities in COVID-19 outcomes remains unclear.

**Objective:**

To evaluate the association of race and ethnicity with COVID-19 outcomes and to examine the association between race, ethnicity, COVID-19 outcomes, and socioeconomic determinants.

**Data Sources:**

A systematic search of PubMed, medRxiv, bioRxiv, Embase, and the World Health Organization COVID-19 databases was performed for studies published from January 1, 2020, to January 6, 2021.

**Study Selection:**

Studies that reported data on associations between race and ethnicity and COVID-19 positivity, disease severity, and socioeconomic status were included and screened by 2 independent reviewers. Studies that did not have a satisfactory quality score were excluded. Overall, less than 1% (0.47%) of initially identified studies met selection criteria.

**Data Extraction and Synthesis:**

Preferred Reporting Items for Systematic Reviews and Meta-Analyses (PRISMA) guidelines were followed. Associations were assessed using adjusted and unadjusted risk ratios (RRs) and odds ratios (ORs), combined prevalence, and metaregression. Data were pooled using a random-effects model.

**Main Outcomes and Measures:**

The main measures were RRs, ORs, and combined prevalence values.

**Results:**

A total of 4 318 929 patients from 68 studies were included in this meta-analysis. Overall, 370 933 patients (8.6%) were African American, 9082 (0.2%) were American Indian or Alaska Native, 101 793 (2.4%) were Asian American, 851 392 identified as Hispanic/Latino (19.7%), 7417 (0.2%) were Pacific Islander, 1 037 996 (24.0%) were White, and 269 040 (6.2%) identified as multiracial and another race or ethnicity. In age- and sex-adjusted analyses, African American individuals (RR, 3.54; 95% CI, 1.38-9.07; *P* = .008) and Hispanic individuals (RR, 4.68; 95% CI, 1.28-17.20; *P* = .02) were the most likely to test positive for COVID-19. Asian American individuals had the highest risk of intensive care unit admission (RR, 1.93; 95% CI, 1.60-2.34, *P* < .001). The area deprivation index was positively correlated with mortality rates in Asian American and Hispanic individuals (*P* < .001). Decreased access to clinical care was positively correlated with COVID-19 positivity in Hispanic individuals (*P* < .001) and African American individuals (*P* < .001).

**Conclusions and Relevance:**

In this study, members of racial and ethnic minority groups had higher risks of COVID-19 positivity and disease severity. Furthermore, socioeconomic determinants were strongly associated with COVID-19 outcomes in racial and ethnic minority populations.

## Key Points

**Question:**

Are race and ethnicity–based COVID-19 outcome disparities in the United States associated with socioeconomic characteristics?

**Findings:**

In this systematic review and meta-analysis of 4.3 million patients from 68 studies, African American, Hispanic, and Asian American individuals had a higher risk of COVID-19 positivity and ICU admission but lower mortality rates than White individuals. Socioeconomic disparity and clinical care quality were associated with COVID-19 mortality and incidence in racial and ethnic minority groups.

**Meaning:**

In this study, members of racial and ethnic minority groups had higher rates of COVID-19 positivity and disease severity than White populations; these findings are important for informing public health decisions, particularly for individuals living in socioeconomically deprived communities.

## Teaser

This systematic review and meta-analysis examines the association between race, ethnicity, COVID-19 outcomes, and socioeconomic determinants.

## Introduction

As of August 19, 2021, more than 209 million people across the world had been infected by COVID-19, with the United States accounting for more than 36 million cases and 618 000 deaths.^1^ COVID-19 disproportionately affects racial and ethnic minority groups.^2^ To reduce exposure and mortality rates, it is critical to identify the disparities associated with greater occurrences of COVID-19 among different populations.^3^

In a meta-analysis of 50 articles,^4^ it was shown that African American and Asian American patients were at a higher risk of intensive care unit (ICU) admission because of COVID-19 than White patients. A separate meta-analysis examining 45 articles^5^ indicated that race may be associated with worse COVID-19 outcomes because of the increased occurrence of comorbidities in racial and ethnic minority groups. However, these studies did not examine the role of socioeconomic determinants, which disproportionately affect racial and ethnic minority populations. Another study^6^ explored underlying factors for COVID-19 outcomes in racial and ethnic minority groups but did not integrate data from external sources, such as county median income. As such, current meta-analyses lack investigations assessing how socioeconomic determinants may be associated with COVID-19 disease severity in minority populations.

Individual cross-sectional and cohort studies have found that COVID-19 infection rates in racial and ethnic minority groups are associated with low socioeconomic status and income.^7,8^ Specifically, studies have found that there is a positive association between COVID-19 risk and area deprivation index (ADI).^9^ Past studies have also demonstrated that 11.7% of African Americans individuals are uninsured, compared with 7.5% of White individuals, thus potentially leading to more severe disease outcomes because of lack of access to medical care.^10^ Geographic variation may also play a role in COVID-19 disease severity, as rural hospitals and communities often lack resources.^11^ Therefore, it is plausible that these social determinants might be associated with COVID-19 disease severity in racial and ethnic minority populations.

In this study, we examine the associations of race and ethnicity with COVID-19 positivity rates, mortality, hospitalization, and ICU admission in the United States. We then associate these outcomes with various social determinants through adjusted and unadjusted relative risk ratio (RR) and odds ratio (OR) calculations and metaregression analysis. To our knowledge, we are the first to examine social determinants of health in racial disparities of COVID-19 outcomes through a systematic review and meta-analysis, which provides a more accurate understanding than results published in single-site studies.

## Methods

### Database Search and Inclusion Criteria

We conducted a systematic search of studies published from January 1, 2020, to January 6, 2021, in the PubMed, medRxiv, bioRxiv, Embase, and the World Health Organization COVID-19 databases. We used search terms pertaining to COVID-19 and disparities (eMethods 1 in the Supplement) and only included studies that reported data on race and ethnicity as well as the following variables: socioeconomic status, COVID-19 positivity, hospitalization, ICU admission, mortality, and location/geography. All included studies were conducted in the United States.

Two independent reviewers (S.M. and D.J.) screened the titles, abstracts, and full text of each eligible study from the selected databases. Disagreements were resolved through discussion with a third reviewer (Y.L.). We followed the Preferred Reporting Items for Systematic Reviews and Meta-Analyses (PRISMA) guideline for selection of papers in this meta-analysis (eFigure 1 and eMethods 2 in the Supplement). The Joanna Briggs Institute critical appraisal tools were used to assess the quality of evidence from all studies in respect to study design. Studies were not included in our analysis if they scored lower than a 6 of 8 (75%) for cohort studies and 9 of 11 (82%) for cross-sectional studies. The complete search and inclusion strategy can be found in the eMethods 1 and 2 in the Supplement.

### Data Extraction

Data were extracted from the 68 studies screened with the PRISMA guidelines. We collected details from studies regarding study setting and type and patient demographic characteristics, comorbidities, and outcomes (eMethods 1 in the Supplement), using the same independent reviewer design as during study selection. Following the initial data review, socioeconomic variables quantifying disparities in health, income, and geography were extracted from external sources using zip code and congressional district location (eMethods 1 in the Supplement).^12,13,14,15^ External measures of socioeconomic disparities were not extracted for studies that occurred at a statewide level or that included data from patients across the United States, as the specific tools we used to determine these values were limited by units of analysis at the county, congressional district, and/or geographic address level.

COVID-19 has been strongly associated with lower socioeconomic status in racial/ethnic minorities.^16^ Accordingly, ADI was used as a quantitative measure of socioeconomic disadvantage, and it accounts for several factors, such as income, education, employment, and housing quality. The Urban Core Opportunity Index (UOI) measures the urbanicity of geographic location, through the characterization of factors such as the amount of renters and households without vehicles.^15^

We also examined the association of clinical care quality with COVID-19 positivity, mortality, ICU admission, and hospitalization through metaregression analysis. Specifically, we investigated the following measures of clinical care quality: preventable hospital stays, ratio of the population to primary care physicians, and percentage of uninsured individuals. A higher rate of preventable hospital stays represents a lower quality of available medical care, and a higher ratio of the population to physicians refers to a larger population with access to only 1 primary care physician.^14^

### Statistical Analysis

All data analysis was conducted using R Studio version 4.1.1 (R Project for Statistical Computing). Analyses were conducted separately for each racial and ethnic group in the following cohorts: COVID-19 positivity, ICU admission, hospitalization, and mortality. Studies with missing data for a particular cohort or variable were excluded from the respective analysis. The following analyses were conducted to investigate the association of race and ethnicity with COVID-19 outcomes. Combined prevalence refers to the incidence of COVID-19 outcomes in a certain population per 1000 patients. Metaregression analysis was conducted to assess associations between study effect size and socioeconomic variables extracted by study location. Relative risk ratios (RRs) and odds ratios (ORs) were also used to assess the associations of race and ethnicity with COVID-19 outcomes, with White individuals as the reference group. Both RR and OR values were adjusted for several key confounders using a linear mixed-effect model (eMethods 3 in the Supplement). Statistical significance was set at *P* < .05, and all tests were 2-tailed. The Egger test was used to assess publication bias, with *P* < .05 as the level of statistical significance (eFigures 2-4 in the Supplement). Information for all the studies is reported in detail in eTables 1 and 2 in the Supplement.

## Results

### Study Characteristics

**Figure 1.**
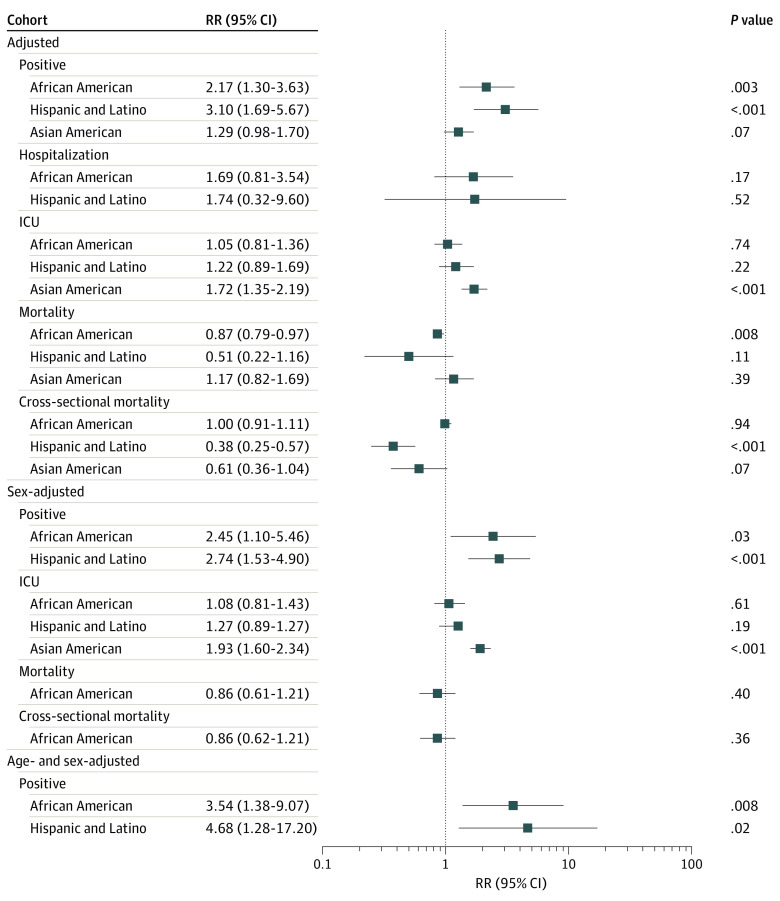
Adjusted, Sex-Adjusted, and Sex- and Age-Adjusted Risk Ratios (RRs) for White, African American, Hispanic, and Asian American Individuals According to COVID-19 Positivity, Hospitalization, Intensive Care Unit (ICU) Admission, and Mortality

**Figure 2.**
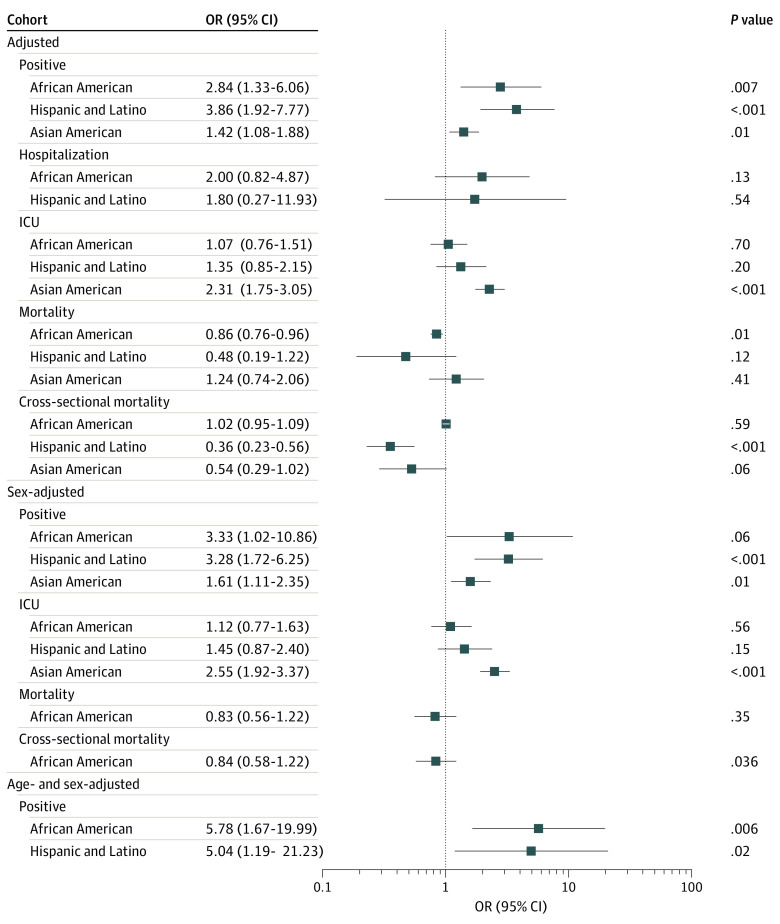
Adjusted, Sex-Adjusted, and Sex- and Age-Adjusted Odds Ratios (ORs) for White, African American, Hispanic, and Asian American Individuals According to COVID-19 Positivity, Hospitalization, Intensive Care Unit (ICU) Admission, and Mortality

A total of 4 318 929 patients from 68 studies^17,18,19,20,21,22,23,24,25,26,27,28,29,30,31,32,33,34,35,36,37,38,39,40,41,42,43,44,45,46,47,48,49,50,51,52,53,54,55,56,57,58,59,60,61,62,63,64,65,66,67,68,69,70,71,72,73,74,75,76,77,78,79,80,81,82,83,84^ were included in this meta-analysis (Table). Overall, 370 933 patients (8.6%) were African American, 9082 (0.2%) were American Indian or Alaska Native, 101 793 (2.4%) were Asian American, 851 392 identified as Hispanic/Latino (19.7%), 7417 (0.2%) were Pacific Islander, 1 037 996 (24.0%) were White, and 269 040 (6.2%) identified as multiracial or of other racial or ethnic group. The studies were separated into cohort and cross-sectional studies for data analysis. All unadjusted and adjusted RR and OR values are reported in Figure 1 and Figure 2 and eTables 3 and 4 in the Supplement.

**Table.** Overall Study Summary Characteristics

Characteristic	No. (%)				
	Included	COVID-19 positivity	Hospitalization	ICU admission	Mortality
**Study characteristics**					
Studies, No.	68	68	6	9	19
Cohort studies	32 (47.1)	32 (47.1)	4 (66.7)	4 (44.4)	10 (52.6)
Cross-sectional studies	36 (52.9)	36 (52.9)	2 (33.3)	5 (55.6)	9 (47.4)
**Population characteristics**					
Population, No.	4 318 929	1 697 421	88 283	8456	1 024 431
White	1 037 996 (24.0)	704 668 (41.5)	37 576 (42.6)	2163 (25.6)	338 495 (33.0)
African American	370 933 (8.6)	204 890 (12.1)	35 340 (40.0)	2195 (25.9)	106 864 (10.4)
Asian American	101 793 (2.3)	80 756 (4.8)	816 (0.9)	437 (5.2)	56 561 (5.5)
Hispanic	851 392 (19.7)	637 476 (37.5)	15 367 (17.4)	3240 (38.3)	522 511 (51.0)
Pacific Islander	7417 (0.2)	NA^a^	NA^a^	NA^a^	NA^a^
American Indian/Alaskan Native	9082 (0.2)	NA^a^	NA^a^	NA^a^	NA^a^
Multiracial/other	269 040 (6.2)	150 387 (8.6)	20 (0.02)	421 (5.0)	303 (0.03)

Abbreviations: ICU, intensive care unit; NA, not applicable.

^a^ Not tracked due to lack of studies.

### COVID-19 Positivity Rates

In age- and sex-adjusted analyses, we found that African American and Hispanic individuals were significantly more likely to test positive for COVID-19 than White individuals (African American: RR, 3.54; 95% CI, 1.38-9.07; *P* = .008; Hispanic: RR, 4.68; 95% CI, 1.28-17.20, *P* = .02) (Figure 1). There was a lack of data to calculate age- and sex-adjusted RR and OR values for Asian American individuals. Following adjustment for ADI, African American and Hispanic individuals were almost 2 times as likely to test positive for COVID-19 as White individuals (African American: RR, 2.01; 95% CI, 1.04-3.88; *P* = .04; Hispanic: RR, 2.09; 95% CI, 1.13-3.88; *P* = .02), followed by Asian American individuals (RR, 1.12; 95% CI, 1.04-1.21; *P* = .003) (Figure 1). After adjustment for clinical care quality, we found that African American individuals were still the most likely to test positive for COVID-19 (RR, 1.79; 95% CI, 1.11-3.17; *P* = .03), followed by Asian American individuals (RR, 1.16; 95% CI, 1.03-1.31; *P* = .02) (Figure 1). Hispanic individuals did not exhibit significant results following adjustment for clinical care quality. Interestingly, adjustment for the UOI demonstrated that Asian American individuals face the highest risk of COVID-19 positivity (RR, 1.13; 95% CI, 1.07-1.19, *P* < .001) (eTable 3 in the Supplement). We did not observe significant results in African American and Hispanic individuals following adjustment for UOI. Combined prevalence values demonstrated similar trends, with African American individuals having the highest prevalence of COVID-19 positivity (eFigure 5 in the Supplement). In summary, with some exceptions, adjusting for ADI and clinical care quality significantly decreased the risk of COVID-19 infection in African American and Hispanic individuals when compared with White individuals. However, the risk still remained high in these populations following adjustment.

### Risk of ICU Admission

COVID-19 disease severity was assessed through ICU admission and hospitalization rates among various racial and ethnic groups (eFigure 6 in the Supplement). Following adjustment for sex, Asian American individuals had a significant RR of 1.93 (95% CI, 1.60-2.34; *P* < .001) compared with White individuals (Figure 1).

### Mortality Rates in Cohort and Cross-sectional Studies

The combined prevalence of COVID-19 mortality rates in cohort studies was highest among White individuals (161.12 per 1000 patients), followed by African American individuals (143.99 per 1000 patients), Hispanic/Latino individuals (130.51 per 1000 patients), and Asian American individuals (42.99 per 1000 patients) (eTable 5 in the Supplement). In cross-sectional studies, the combined prevalence of mortality rates were highest among African American individuals (277.15 per 1000 patients), followed by Hispanic individuals (213.34 per 1000 patients), White individuals (173.38 per 1000 patients), and Asian individuals (80.4 per 1000 patients) (eTable 5 and eFigure 7 in the Supplement).

The ADI-adjusted RR for cross-sectional studies found that Hispanic individuals were at a lower risk of COVID-19 mortality than White individuals (RR, 0.44; 95% CI, 0.31-0.61; *P* < .001). Similarly, the county median income–adjusted RR showed that Hispanic and Asian American individuals were at a lower risk of COVID-19 mortality than White individuals (Hispanic: RR, 0.43; 95% CI, 0.41-0.46; *P* < .001; Asian American: RR, 0.44; 95% CI, 0.36-0.54; *P* = .001) (Figure 1).

### ADI and Racial Disparities in COVID-19 Mortality

**Figure 3.**
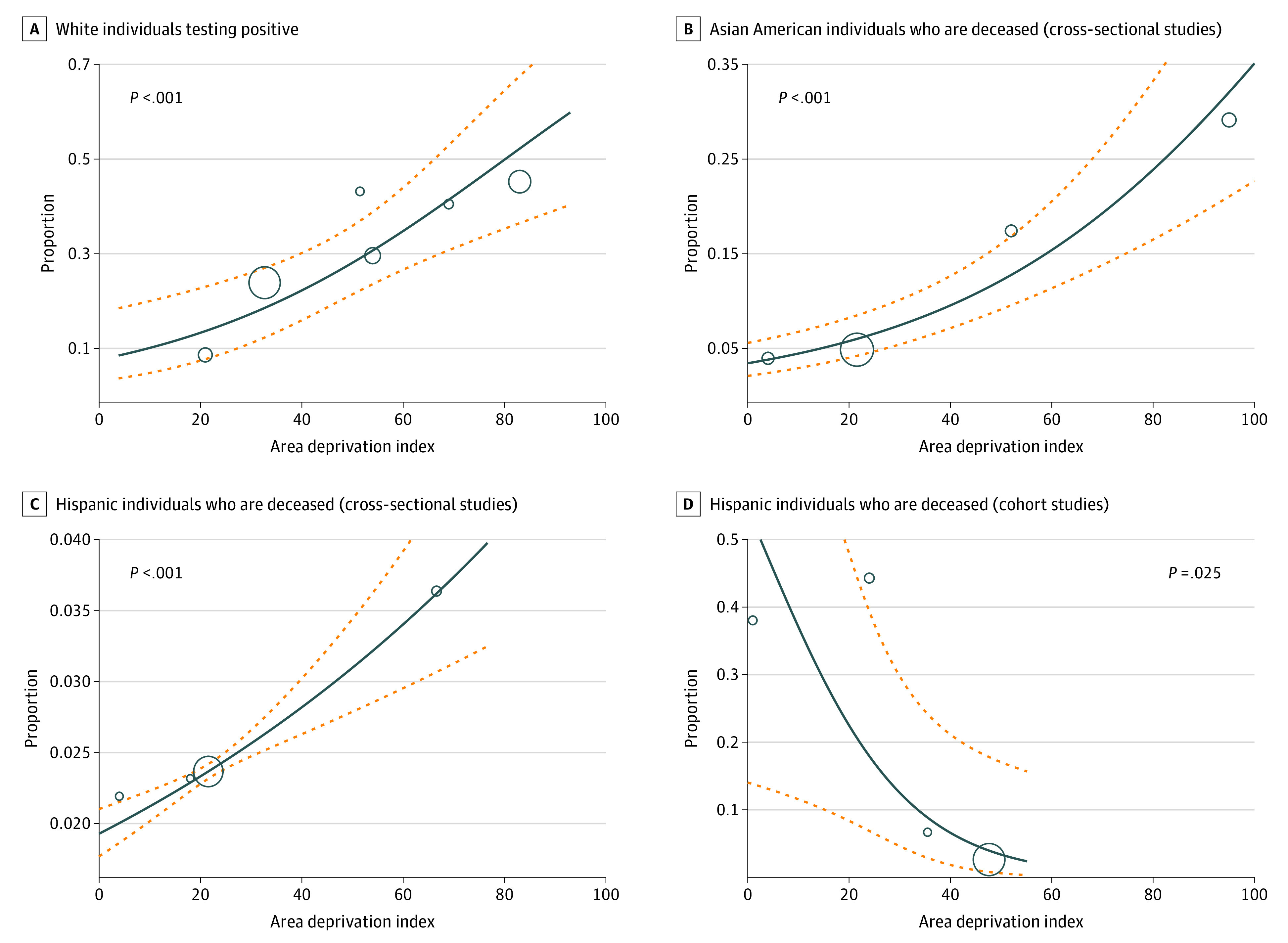
Metaregression of Area Deprivation Index in White Individuals Who Tested Positive for COVID-19 and Asian American and Hispanic Individuals Who Died From COVID-19 The solid line represents the association between the 2 variables. The dashed lines represent the 95% CI. The circles represent the different studies this particular graph is accounting for, while the sizes of the circles represent the weight of each of the studies.

We further investigated the association of ADI with COVID-19 positivity and disease severity by race and ethnicity through metaregression analysis. A higher ADI corresponds to worse socioeconomic status. Accordingly, we found that an increase in ADI was positively associated with the mortality rates of Asian American and Hispanic individuals in cross-sectional studies (*P* < .001) (Figure 3). Interestingly, an increase in ADI was negatively associated with mortality rates of Hispanic individuals in cohort studies (*P* = .03) (Figure 3).

### Metaregression With County Median Income

**Figure 4.**
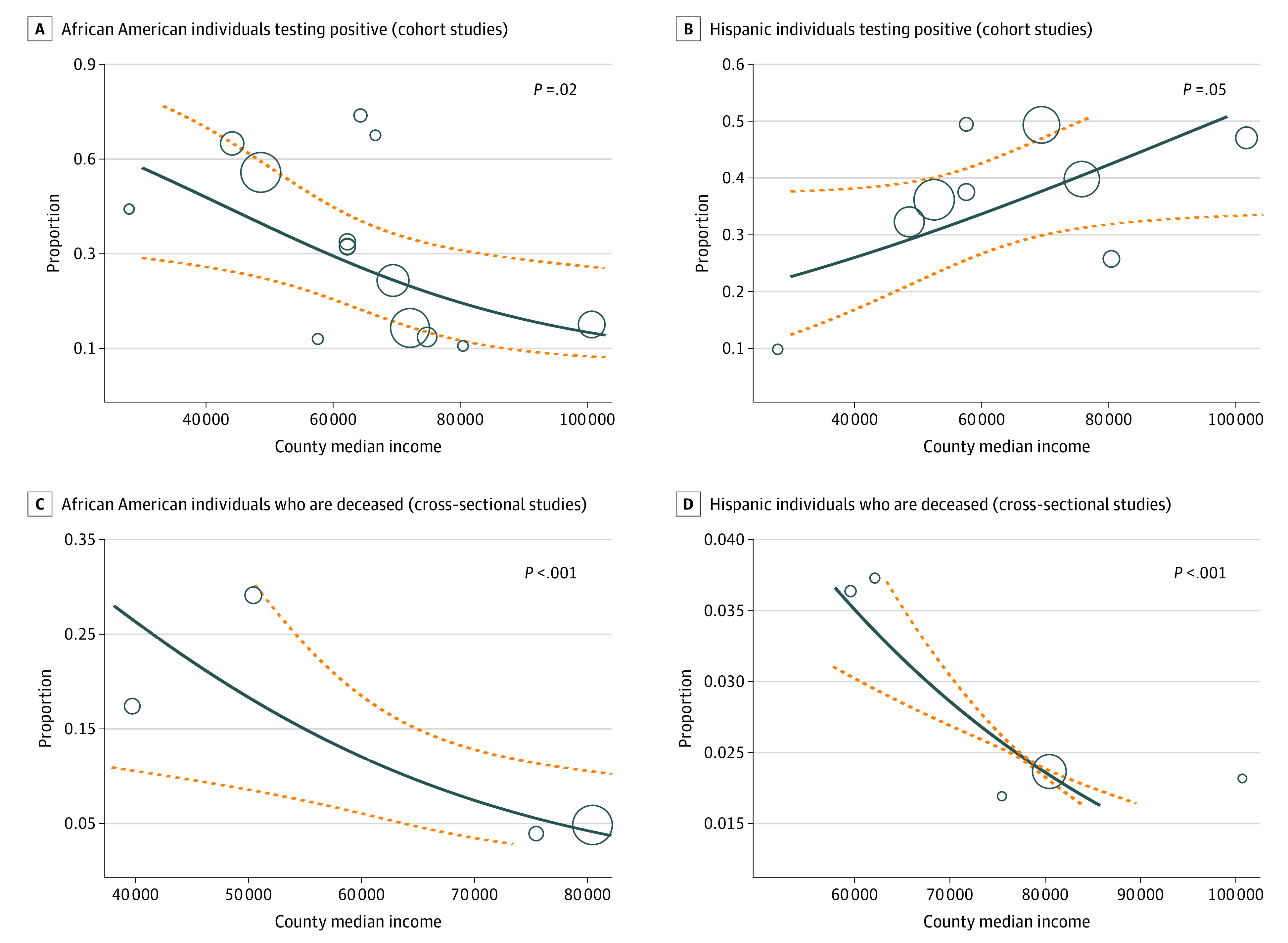
Metaregression of County Median Income in African American and Hispanic Individuals Who Tested Positive for COVID-19 and African American and Hispanic Individuals Who Died From COVID-19 The solid line represents the association between the 2 variables. The dashed lines represent the 95% CI. The circles represent the different studies this particular graph is accounting for, while the sizes of the circles represent the weight of each of the studies.

We conducted metaregression analysis to assess the association of county median income with COVID-19 outcomes by race and ethnicity. Although ADI is a more comprehensive measure of socioeconomic deprivation, we also analyzed county median income because it provided more significant results for RR/OR adjustment in comparison with ADI. Therefore, we determined that we should further examine any associations with income, as it may have been more strongly associated with COVID-19 outcomes than other socioeconomic measures included in ADI. In cohort studies, we found that county median income was negatively associated with mortality rates in Asian American populations (*P* < .001). In cross-sectional studies, higher county median income was associated with lower mortality rates in Hispanic and African American individuals (*P* < .001). County median income was also negatively associated with the proportion of White individuals admitted to the ICU (*P* = .02) (Figure 4; eFigure 8 in the Supplement).

Through further metaregression analysis, we determined that Hispanic individuals had a positive association of increasing income and positivity rates (*P* = .03). However, African American individuals displayed a negative association between income and positivity rates (*P* = .02).

We additionally conducted Spearman correlations to assess the degree of association between these studied determinants. We observed a strong, positive correlation between county median income and area deprivation index (*R* = 0.61; *P* < .001), as county median income was among the measures included in developing the ADI (eFigure 9 in the Supplement). We found there was a lesser degree of association between county median income and measures of clinical care quality (eFigure 9 in the Supplement).

### Metaregression With Clinical Care Variables

In cohort studies, we found that an increase in number of preventable hospital stays (*P* = .04) and the population served by 1 primary care physician (*P* = .009) were associated with a decrease in positivity among Asian American individuals (eFigure 10 in the Supplement). Conversely, the population served by 1 primary care physician was positively associated with COVID-19 positivity among Hispanic individuals (*P* < .001). In cross-sectional studies, we found that the ratio of the population served to primary care physicians was positively correlated with mortality among White individuals (*P* < .001). The percentage of uninsured individuals was positively associated with positivity among African American (*P* < .001) and White (*P* = .01) individuals in cohort and cross-sectional studies.

### Risk of Bias Across Studies

We found that cohort studies detailing the proportion of Asian American and Hispanic individuals who tested positive for COVID-19, cohort studies detailing the proportion of Asian American individuals admitted to the ICU, cross-sectional studies detailing the proportion of Asian American individuals who died, and cohort studies detailing the proportion of African American individuals who died exhibited publication bias. To evaluate the association of study heterogeneity with summary proportions, we conducted leave-one-out sensitivity analysis to measure the effects of outliers (eTable 6 and eFigures 11-13 in the Supplement). We found that summary proportions were not significantly altered by the removal of these outliers (eFigures 14 and 15 in the Supplement). However, we observed that following the removal of the outlier in the COVID-19 mortality group (ie, cohort studies), African American individuals had the highest rate of mortality followed by Asian American individuals. Prior to removal of the outlier, we found that mortality rates were highest among White individuals. We additionally observed high heterogeneity statistics in our results, indicating that there may be variability in the studies included.

## Discussion

In our meta-analysis, we found that COVID-19 positivity and ICU admission rates were higher in African American, Hispanic, and Asian American individuals compared with White individuals, with some exceptions. Our results are consistent with previous findings that suggest that racial and ethnic minority groups face a higher risk of ICU admission and COVID-19 positivity but a lower risk of mortality than White populations.^65,85,86,87,88,89^

However, current meta-analyses do not provide associations with socioeconomic variables, which are highly implicated in COVID-19 outcomes. Therefore, in this study, we aimed to investigate both racial and ethnic disparities in COVID-19 outcomes as well as their associations with socioeconomic variables.

Following adjustment for ADI and clinical care quality, we found that risk of COVID-19 positivity in African-American and Hispanic individuals substantially decreased. However, the risk for COVID-19 positivity following adjustment remained higher in these minoritized populations when compared with Whites. As such, this occurrence may be because of the overrepresentation of members of racial and ethnic minority groups in essential jobs, which increase exposure to COVID-19. Furthermore, comorbidities, such as hypertension or obesity, are prevalent among minority populations, thus contributing to worsened disease outcomes.^8,90,91^ To our knowledge, we are the first to adjust RRs and ORs of race-associated COVID-19 outcomes using health care quality and access.

We further examined the association of socioeconomic determinants with COVID-19 positivity rates, mortality rates, hospitalization, and ICU admission in racial and ethnic minority groups through metaregression analysis. Increased deprivation was found to be associated with increased mortality in Asian American individuals. Paradoxically, an increase in county median income was associated with increased mortality rates in Asian American individuals. This result suggests that factors other than income that contribute to ADI, such as education, housing equality, and employment, could affect Asian American populations. One hypothesis is that a large number of Asian American individuals work in health care settings, which can lead to increased mortality rates that do not reflect the general population of the surrounding community.^92^

An increase in deprivation was also found to be associated with decreased mortality rates in Hispanic individuals in cohort studies, although the opposite result was seen in cross-sectional studies. This inconsistency suggests that further research is needed to establish conclusively the association between mortality rates and deprivation in Hispanics.

We additionally assessed associations between measures of clinical care quality and COVID-19 outcomes. Curiously, we found that an increase in preventable hospital stays and the population served by 1 primary physician were associated with a decrease in the percentage of Asian American individuals who tested positive for COVID-19, suggesting again that other variables may be affecting COVID-19 positivity rates in this population.

Conversely, we observed a positive association between lack of primary care physician access (ie, increased ratio of population to physician) and COVID-19 positivity among Hispanic individuals. Past studies have demonstrated that Hispanic individuals are less likely to delay care if the primary care physician to patient ratio is improved.^93^

An increase in the number of uninsured individuals was also positively associated with COVID-19 positivity among African American individuals. African American individuals are less likely to have health insurance coverage compared with White individuals.^94^ Members of racial and ethnic minority groups who are uninsured may also not have access to COVID-19 tests.^10^

Collectively, our findings demonstrate that racial and ethnic minority groups have faced higher risk of COVID-19 positivity and ICU admission. Public health policies should address socioeconomic and racial disparities to reduce exposure to and fatality from COVID-19 in underrepresented populations. Increasing equitable access to health care and improving resources for underserved populations may reduce exposure to COVID-19 in racial/ethnic minorities.

### Limitations

Our study has several limitations. First, we found high heterogeneity statistics, indicating that there may be variation in the effect sizes of the studies. Second, a number of publications that were included had incomplete or missing data on mortality, positivity, ICU admission, and hospitalization rates. Moreover, there were limited data on several racial and ethnic groups. There was also a lack of information on comorbidities in some studies, which limited our ability to adjust for these variables. Additionally, several study cohorts exhibited publication bias. As publication bias reduces the accuracy of results, the validity of results in these particular study cohorts may be limited.

## Conclusions

In this study, African American, Hispanic, and Asian American individuals were at considerably higher risk of COVID-19 positivity and ICU admission compared with White individuals. Adjustment for social determinants of health and socioeconomic factors decreased risks of COVID-19 positivity in racial and ethnic minority groups; however, several factors were not accounted for by these variables. We also observed that decreased access to clinical care was positively associated with COVID-19 positivity in Hispanic and African American individuals. In conclusion, we found that racial and ethnic disparities in COVID-19 outcomes could be accounted for by socioeconomic determinants in some populations, such as African American, Hispanic, and Asian American individuals.

## Notes

Supplement. **eTable 1. **Detailed Study Summary Characteristics of All 68 Included Studies **eTable 2. **Study Summary Characteristics for Comorbidities **eTable 3. **Adjustment of Relative Risk Ratios (RRs) for Additional Variables **eTable 4. **Adjustment of Odds Ratios (ORs) for Additional Variables **eTable 5. **Combined Prevalence of Cohort and Cross-sectional Studies **eTable 6. **Summary of Q and *I^2^* Statistics for Study Variables **eFigure 1. **PRISMA Workflow for Studies Included in Analysis **eFigure 2. **Funnel Plots for Deceased Individuals in Cohort and Cross-sectional Studies **eFigure 3. **Funnel Plots for Patients Admitted to ICU or Hospitalized in Cohort Studies **eFigure 4. **Funnel Plots for COVID-19 Positive Patients in Cohort and Cross-sectional Studies **eFigure 5. **Forest Plot for COVID-19 Positivity in Cohort and Cross-sectional Studies **eFigure 6. **Forest Plot for Patients Admitted to ICU or Hospitalized in Cohort Studies **eFigure 7. **Forest Plot for Deceased Individuals in Cohort and Cross-sectional Studies **eFigure 8.** Metaregression for County Median Income **eFigure 9.** Spearman Correlations for Measures of Clinical Care Quality **eFigure 10. **Metaregression for Clinical Care Measures **eFigure 11.** Leave-One-Out Sensitivity Analysis for Deceased Individuals in Cohort and Cross-sectional Studies **eFigure 12.** Leave-One-Out Sensitivity Analysis for Patients Admitted to ICU or Hospitalized in Cohort Studies **eFigure 13. **Leave-One-Out Sensitivity Analysis for COVID-19 Positive Patients in Cohort and Cross-sectional Studies **eFigure 14. **Forest Plots for Deceased Patients After Removing Dominating Studies **eFigure 15.** Forest Plots for Positive Individuals After Removing Dominating Studies **eMethods 1. **Methods Pertaining to Search Criteria and Data Collection **eMethods 2.** Citations of Articles that Appeared to Meet Inclusion Criteria but Were Excluded **eMethods 3.** Description of Statistical Methods Used in Analyses
