# Error in Figures 1 and 2

**DOI:** 10.1001/jamanetworkopen.2021.44237

**Published:** 2021-12-21

In the Original Investigation titled “Disparities in COVID-19 Outcomes by Race, Ethnicity, and Socioeconomic Status: A Systematic Review and Meta-analysis,”^1^ published November 11, 2021, there were errors in Figures 1 and 2. The odds ratios for the first model in each figure were erroneously labeled as adjusted; in fact, they were unadjusted. This article has been corrected.^1^

## References

[1] Magesh S, John D, Li WT, . Disparities in COVID-19 outcomes by race, ethnicity, and socioeconomic status: a systematic review and meta-analysis. JAMA Netw Open. 2021;4(11):e2134147. doi:10.1001/jamanetworkopen.2021.34147 34762110 PMC8586903

