# Error in Interpretation of Mortality

**DOI:** 10.1001/jamanetworkopen.2022.2170

**Published:** 2022-02-24

The Original Investigation titled “Disparities in COVID-19 Outcomes by Race, Ethnicity, and Socioeconomic Status: A Systematic Review and Meta-analysis,”^1^ published November 11, 2021, has been corrected to remove the interpretation of mortality risk estimates from unadjusted regression models. The corrections appear in the Key Points, Results, and Discussion sections. The authors provided a Comment describing these changes.^2^ The article was previously corrected to address errors in figure titles.^3^
