# Supplementary material for: Disparities in COVID-19 Outcomes by Race, Ethnicity, and Socioeconomic Status: A Systematic-Review and Meta-analysis
Source: JAMA Netw Open. 2021 Nov 11;4(11):e2134147. doi: 10.1001/jamanetworkopen.2021.34147 (PMC8586903; doi:10.1001/jamanetworkopen.2021.34147)
Supplement: Supplement. — eTable 1. Detailed Study Summary Characteristics of All 68 Included Studies eTable 2. Study Summary Characteristics for Comorbidities eTable 3. Adjustment of Relative Risk Ratios (RRs) for Additional Variables eTable 4. Adjustment of Odds Ratios (ORs) for Additional Variables eTable 5. Combined Prevalence of Cohort and Cross-sectional Studies eTable 6. Summary of Q and I2 Statistics for Study Variables eFigure 1. PRISMA Workflow for Studies Included in Analysis eFigure 2. Funnel Plots for Deceased Individuals in Cohort and Cross-sectional Studies eFigure 3. Funnel Plots for Patients Admitted to ICU or Hospitalized in Cohort Studies eFigure 4. Funnel Plots for COVID-19 Positive Patients in Cohort and Cross-sectional Studies eFigure 5. Forest Plot for COVID-19 Positivity in Cohort and Cross-sectional Studies eFigure 6. Forest Plot for Patients Admitted to ICU or Hospitalized in Cohort Studies eFigure 7. Forest Plot for Deceased Individuals in Cohort and Cross-sectional Studies eFigure 8. Metaregression for County Median Income eFigure 9. Spearman Correlations for Measures of Clinical Care Quality eFigure 10. Metaregression for Clinical Care Measures eFigure 11. Leave-One-Out Sensitivity Analysis for Deceased Individuals in Cohort and Cross-sectional Studies eFigure 12. Leave-One-Out Sensitivity Analysis for Patients Admitted to ICU or Hospitalized in Cohort Studies eFigure 13. Leave-One-Out Sensitivity Analysis for COVID-19 Positive Patients in Cohort and Cross-sectional Studies eFigure 14. Forest Plots for Deceased Patients After Removing Dominating Studies eFigure 15. Forest Plots for Positive Individuals After Removing Dominating Studies eMethods 1. Methods Pertaining to Search Criteria and Data Collection eMethods 2. Citations of Articles that Appeared to Meet Inclusion Criteria but Were Excluded eMethods 3. Description of Statistical Methods Used in Analyses [file jamanetwopen-e2134147-s001.pdf]

Supplemental Online Content

Magesh S, John D, Li WT, et al. Disparities in COVID-19 outcomes by race, ethnicity, and socioeconomic status. *JAMA Netw Open.* 2021;4(11):e2134147. doi:10.1001/jamanetworkopen.2021.34147

eTable 1. Detailed Study Summary Characteristics of All 68 Included Studies

eTable 2. Study Summary Characteristics for Comorbidities

eTable 3. Adjustment of Relative Risk Ratios (RRs) for Additional Variables

eTable 4. Adjustment of Odds Ratios (ORs) for Additional Variables

eTable 5. Combined Prevalence of Cohort and Cross-sectional Studies

eTable 6. Summary of Q and I^2 Statistics for Study Variables

eFigure 1. PRISMA Workflow for Studies Included in Analysis

eFigure 2. Funnel Plots for Deceased Individuals in Cohort and Cross-sectional Studies

eFigure 3. Funnel Plots for Patients Admitted to ICU or Hospitalized in Cohort Studies

eFigure 4. Funnel Plots for COVID-19 Positive Patients in Cohort and Cross-sectional Studies

eFigure 5. Forest Plot for COVID-19 Positivity in Cohort and Cross-sectional Studies

eFigure 6. Forest Plot for Patients Admitted to ICU or Hospitalized in Cohort Studies

eFigure 7. Forest Plot for Deceased Individuals in Cohort and Cross-sectional Studies

eFigure 8. Metaregression for County Median Income

eFigure 9. Spearman Correlations for Measures of Clinical Care Quality

eFigure 10. Metaregression for Clinical Care Measures

eFigure 11. Leave-One-Out Sensitivity Analysis for Deceased Individuals in Cohort and Cross-sectional Studies

eFigure 12. Leave-One-Out Sensitivity Analysis for Patients Admitted to ICU or Hospitalized in Cohort Studies

eFigure 13. Leave-One-Out Sensitivity Analysis for COVID-19 Positive Patients in Cohort and Cross-sectional Studies

eFigure 14. Forest Plots for Deceased Patients After Removing Dominating Studies

eFigure 15. Forest Plots for Positive Individuals After Removing Dominating Studies

eMethods 1. Methods Pertaining to Search Criteria and Data Collection

eMethods 2. Citations of Articles that Appeared to Meet Inclusion Criteria but Were Excluded

eMethods 3. Description of Statistical Methods Used in Analyses

© 2021 Magesh S et al. *JAMA Network Open.*

This supplemental material has been provided by the authors to give readers additional information about their work.

© 2021 Magesh S et al. *JAMA Network Open.*

eTable 1. Detailed Study Summary Characteristics of All 68 Included Studies

Summary characteristics for each of the 68 studies included in this systematic review and meta-analysis, including but not limited to the following variables: ADI, county median income, measures of clinical care quality, and urban core opportunity index.

Study First Author	Study Type	Quality Score	Geographic Region	Number of patients (total)	African-American	White	Asian-American	Pacific Islander	Hispanic /Latino	American Indian/Alaskan Natives	multiracial	Mean Age	Median Age	Study Setting	ADI	Congressional County	County Median Income	Percent Uninsured	Primary Care Physicians (X:1)	Mental Health Providers (X:1)	Preventable Hospital Stays	UOI	Percent of Individuals Over Age 60	Percent of Individuals Over Age 65	Percent Male
David P. Bui et al, 2020	Cohort	75%	USA	1305130	4086	3337	236	378	4086	322	0	N/A	0	Community	N/A	N/A	N/A	N/A	N/A	N/A	N/A	N/A	N/A	N/A	N/A
Di Xiong et al, 2020	Cross-Sectional	82%	California	848166	0	104140	33342	0	366314	0	0	N/A	N/A	Community	21.56	N/A	80440	8	1250	270	3358	N/A	15.67	10.66	N/A
Erin K. Stokes et al, 2020	Cross-Sectional	88%	USA	599636	131920	215869	23985	5996	197880	7795	23985	N/A	N/A	Community	N/A	N/A	N/A	N/A	N/A	N/A	N/A	N/A	N/A	N/A	48.9
Ishaan Pathak et al, 2020	Cross-Sectional	82%	USA	577117	53495	103554	19637	0	179997	0	220434	N/A	N/A	Community	47.637	N/A	69423	8	1263.3	353.33	4390.7	N/A	N/A	8.27	N/A
Mary L. Adams et al, 2020	Cross-Sectional	88%	USA	398865	31939	307282	9108	0	26837	0	0	N/A	N/A	Community	N/A	N/A	N/A	N/A	N/A	N/A	N/A	N/A	36.6	N/A	56.3
Michael Poulson et al, 2020	Cross-Sectional	100%	USA	124780	48334	76426	0	0	0	0	0	N/A	N/A	Community	N/A	N/A	N/A	N/A	N/A	N/A	N/A	N/A	N/A	N/A	N/A
Sara J. Cromer et al, 2020	Cohort	100%	Mass Gen Brigham - Massachusetts	57865	5528	34398	2077	0	12358	0	0	N/A	N/A	Hospital	18	MA-8	100690	3.66	1423	165	5353.3	1.387	N/A	29.29	44.22
Jon Zelner et al, 2020	Cross-Sectional	82%	Michigan	49719	19662	23301	1364	0	3657	123	1612	N/A	N/A	Community	N/A	N/A	N/A	N/A	N/A	N/A	N/A	N/A	0	N/A	N/A
Diego A. Martinez et al, 2020	Cohort	75%	Baltimore – Washington and DC	37727	11639	17113	0	0	4169	0	0	N/A	N/A	Hospital	35.5	N/A	77674	N/A	N/A	N/A	N/A	N/A	N/A	N/A	N/A
Yelena Rozenfeld et al, 2020	Cross-Sectional	75%	Providence Health System - 5 US States	34503	1649	24799	1713	356	3565	465	1956	N/A	N/A	Hospital	N/A	N/A	N/A	N/A	N/A	N/A	N/A	N/A	33.8	N/A	40.4
Jacob McPadden et al, 2020	Cohort	82%	Yale New Haven Health - New Haven	28605	5093	16825	721	79	5468	60	4464	N/A	N/A	Hospital	N/A	CT-3	70574	7	1140	256.67	4021	4.574	N/A	22.6	39.9
Samuel B Reichberg et al, 2020	Cohort	100%	Northwell Health Laboratories - New York	26730	4534	11463	1572	*	*	*	*	N/A	N/A	Hospital	32.7	N/A	72108	5	700	320	3422	2.6768668	N/A	N/A	N/A
Farhaan S Vahidy et al, 2020	Cross-Sectional	88%	Houston	20228	4369	16648	1861	0	3600	0	263	51	N/A	Community	15	TX-9	49147	N/A	N/A	N/A	N/A	N/A	N/A	N/A	38
Sharia M Ahmed et al, 2020	Cross-Sectional	88%	University of Utah Health Clinics	20088	361	13151	446	223	2804	152	0	N/A	N/A	Hospital	N/A	UT-2	80119	12	2280	566.43	3103.8	1.391	16.3	N/A	44
Kristen M. J. Azar et al, 2020	Cohort	73%	Sutter Health - Northern California	14036	940	6779	1432	98	2681	0	0	50.7	N/A	Hospital	21.56	N/A	80440	N/A	N/A	N/A	N/A	N/A	32	N/A	39.3
Alan Pan et al, 2020	Cross-Sectional	100%	Houston Methodist Hospital	12084	3049	3346	0	0	4372	0	0	N/A	N/A	Hospital	15	N/A	52540	0	0	0	0	0	N/A	N/A	N/A
Lara Jehi et al, 2020	Cohort	100%	Cleveland Clinic - Ohio and Florida	11672	2345	7848	183	0	553	0	1296	N/A	N/A	Hospital	N/A	N/A	N/A	N/A	N/A	N/A	N/A	N/A	N/A	N/A	N/A
Baligh R Yehia et al, 2020	Cross-Sectional	75%	92 Hospitals across 12 US States	11210	4180	4606	0	0	0	0	2424	N/A	61	Hospital	N/A	N/A	N/A	N/A	N/A	N/A	N/A	N/A	0	N/A	49.8
George N. Ioannou et al, 2020	Cohort	100%	USA	10131	4215	5022	80	0	944	140	0	N/A	N/A	Hospital	N/A	N/A	N/A	N/A	N/A	N/A	N/A	N/A	N/A	51	91
Gbenga Ogedegbe et al, 2020	Cohort	73%	Health System in New York	9722	1353	4187	831	0	2087	0	1264	N/A	N/A	Hospital	N/A	N/A	N/A	N/A	N/A	N/A	N/A	6.1547232	N/A	21.6	41.2
Rafi Kabarriti et al, 2020	Cohort	82%	Montefiore Medical Center - New York	9268	2823	960	283	0	2919	0	2283	N/A	N/A	Hospital	21	NY-13	46298	7.5	1185	460	4210	2.644	43	N/A	46.9
Michael Gottlieb et al, 2020	Cross-Sectional	100%	Rush University Medical Center - Chicago, Illinois	8673	2301	1797	119	0	4281	0	4456	N/A	41	Hospital	33.66	N/A	69429	8	1400	540	5350	2.72	N/A	10	46.6

© 2021 Magesh S et al. *JAMA Network Open.*

Brian E. Dixon et al, 2020	Cohort	82%	Indiana	8214	0	6596	0	0	649	0	0	N/A	N/A	Community	69	N/A	57603	10	1500	590	4795	N/A	34.1	N/A	44.4
Haotian Chen et al, 2020	Cross-Sectional	82%	Chicago	8208	2702	2238	140	14	4772	15	0	N/A	40	Community	33.66	N/A	69429	8	1400	540	5350	2.72	N/A	N/A	46.45
Fatima Rodriguez et al, 2020	Cross-Sectional	75%	USA	7868	2009	2768	498	0	2593	0	0	N/A	63	Hospital	N/A	N/A	N/A	N/A	N/A	N/A	N/A	N/A	0	N/A	55
Ahmad Khan et al, 2020	Cohort	82%	USA	7082	3329	2620	0	0	0	0	0	54.1	N/A	Hospital	N/A	N/A	N/A	N/A	N/A	N/A	N/A	N/A	N/A	22.83	38.42
Sara Y. Tartof et al, 2020	Cross-Sectional	100%	Kaiser Permanente Health - Southern California	6916	584	1210	1036	0	3751	0	335	49.1	49	Hospital	N/A	N/A	N/A	9	1565.7	354.28	3589.42	N/A	24.8987	N/A	44.98
Michelle A Waltenburg et al, 2020	Cross-Sectional	100%	28 US States	5721	362	963	0	232	4164	0	0	N/A	N/A	Community	N/A	N/A	N/A	N/A	N/A	N/A	N/A	N/A	N/A	N/A	N/A
Tian Gu et al, 2020	Cohort	100%	University of Michigan Hospital	5698	1058	3740	0	0	0	0	900	53	53	Hospital	15	MI-12	62253	6	570	180	3289	2.8465316	N/A	28.2	46.6
Nicholas E. Ingraham et al, 2020	Cohort	82%	Midwest	5577	172	381	161	0	102	0	0	N/A	60.9	Hospital	N/A	N/A	N/A	N/A	N/A	N/A	N/A	N/A	N/A	N/A	49.1
S.B. Chan et al, 2020	Cohort	82%	Amita Health Center - Chicago, Illinois	5489	1098	2080	0	0	1839	0	472	N/A	N/A	Hospital	N/A	N/A	N/A	N/A	N/A	N/A	N/A	N/A	N/A	N/A	N/A
Kengo Inagaki et al, 2020	Cohort	75%	Mississippi Medical Center	4802	2975	1257	0	0	203	0	0	N/A	N/A	Hospital	37	MS-03	49863	13	1230	310	5433	N/A	0	0	49.4
Ayodeji Adegunsoye et al, 2020	Cohort	75%	Chicago	4413	2543	1071	184	6	254	5	0	52	N/A	Hospital	44	IL-01	56680	10	1050	340	5144	2.248011 4	N/A	N/A	20.1
Nir Menachemi et al, 2020	Cohort	75%	Indiana	3658	0	3373	0	0	80	0	281	N/A	N/A	Community	N/A	N/A	N/A	N/A	N/A	N/A	N/A	N/A	36	N/A	2.13
Eboni G Price-Haywood et al, 2020	Cross-Sectional	83%	Ochsner Health Facility - Louisiana	3481	2451	1030	0	0	0	0	0	N/A	N/A	Hospital	N/A	N/A	N/A	N/A	N/A	N/A	N/A	N/A	N/A	N/A	40
Heather E. Hsu et al, 2020	Cross-Sectional	100%	Urban Safety-Net Medical Center - Boston, Massachusetts	2729	1218	369	0	0	821	0	0	N/A	N/A	Hospital	4	MA-7	75461	4	670	120	5131	3.0717478	34	N/A	48.1
Amy K Feehan et al, 2020	Cohort	75%	New Orleans, Louisiana	2640	0	1631	0	0	0	0	0	50.6	N/A	Community	51.5	N/A	27915	11	1025	275	4848.5	N/A	N/A	N/A	36.5
L. Silvia Muñoz-Price et al, 2020	Cross-Sectional	88%	Froedtert & MCW Hospital - Wisconsin	2595	785	0	0	0	0	0	1810	54.5	N/A	Hospital	N/A	N/A	N/A	8	1360	330	5196	1.0133957	40.1	N/A	46.1
Rolando G. Valenzuela et al, 2020	Cross-Sectional	100%	Stony Brook Hospital - NYC	2039	0	1079	0	0	960	0	0	N/A	N/A	Hospital	N/A	N/A	N/A	5	1380	360	4251	2.2302895	30.22	N/A	46.27
Naima T. Joseph et al, 2020	Cohort	75%	Atlanta	1882	1353	226	164	0	139	0	0	N/A	N/A	Hospital	N/A	N/A	60247	14	900	410	4273	2.0589325 5	N/A	N/A	N/A
Olga Grechukhina et al, 2020	Cross-Sectional	82%	3 Hospitals in New Haven	1567	0	0	0	0	61	0	0	0	0	Hospital	32	CT-03	70574	0	0	0	0	0	0	0	0
Ana A. Weil et al, 2020	Cross-Sectional	75%	16 Skilled Nursing Homes across Washington	1289	360	360	392	34	109	3	111	50	N/A	Hospital	N/A	N/A	N/A	N/A	N/A	N/A	N/A	N/A	N/A	N/A	23
Jennifer Woo Baidal et al, 2020	Cross-Sectional	82%	14 Midwest hospitals	1022	0	437	0	0	0	0	0	N/A	62.1	Community	20	N/A	46298	N/A	N/A	N/A	N/A	N/A	N/A	N/A	48.6
Beth L. Pineles et al, 2020	Cohort	75%	Houston, Texas	935	125	27	84	0	527	0	0	N/A	N/A	Hospital	N/A	N/A	80922	21	5790	4590	4843	N/A	N/A	N/A	0
Ingrid V Bassett et al, 2020	Cross-Sectional	88%	Massachusetts General Hospital - Massachusetts, USA	866	98	346	0	0	305	0	117	N/A	60.4	Hospital	18	MA-8	100690	3.66	1423	165	5353.3	1.387	0	42	56.6
Brian T. Garibaldi et al, 2020	Cohort	91%	Five hospitals in the Maryland and Washington and DC areas	832	336	264	48	0	134	2	42	N/A	63	Hospital	35.5	N/A	77674	N/A	N/A	N/A	N/A	N/A	N/A	N/A	53

© 2021 Magesh S et al. *JAMA Network Open.*

Benjamin D. Renelus et al, 2020	Cohort	100%	New York-Presbyterian Brooklyn Methodist Hospital	734	372	214	0	0	92	0	56	N/A	N/A	Hospital	1	N/A	66891	N/A	N/A	N/A	N/A	N/A	N/A	N/A	41
Angelico Mendy et al, 2020	Cross-Sectional	75%	University of Cincinnati Health System	689	176	201	0	0	224	0	88	N/A	N/A	Hospital	N/A	N/A	N/A	N/A	N/A	N/A	N/A	N/A	N/A	N/A	N/A
Nana-Yaa Misa et al, 2020	Cohort	100%	Alameda Health System - California	526	214	83	41	0	139	0	0	N/A	N/A	Hospital	17	CA-13	91514	N/A	770	150	3000	1.565	N/A	N/A	N/A
Priyank Shah et al, 2020	Cohort	75%	Phoebe Putney Health System - Southwest Georgia	522	454	59	0	0	0	0	9	N/A	63	Hospital	52	GA-2	39728	17	4208.6	3162	4800	N/A	N/A	N/A	42
Wesley H Self et al, 2020	Cross-Sectional	100%	34 hospitals in USA	479	112	0	0	0	178	0	0	N/A	N/A	Hospital	N/A	N/A	N/A	N/A	N/A	N/A	N/A	N/A	N/A	N/A	55.7
Sindhura Bandi et al, 2020	Cohort	75%	Rush University Medical Center - Illinois	474	205	117	7	0	0	0	0	N/A	N/A	Hospital	N/A	IL-07	64312	8	1400	540	5350	2.72	N/A	N/A	N/A
Ashish Bhargava et al, 2020	Cohort	82%	Tertiary Care Academic Health Center, Detroit	419	419	0	0	0	0	0	0	N/A	N/A	Hospital	95	MI-14	50438	6.5	1045	527.14	4584.6	N/A0.82	58.4	47.9	50.35
Vijay Gayam et al, 2020	Cross-Sectional	75%	Teaching Community Hospitals - NYC	408	408	0	0	0	0	0	0	N/A	N/A	Hospital	N/A	NY-9	69754	8	1480	430	4561	3.140101 9	0	N/A	N/A
Samina Bhumbra et al, 2020	Cohort	82%	Riley Hospital - Indianapolis	407	7	4	1	0	7	0	0	N/A	5	Hospital	28	IN-7	46118	12	1210	330	4873	2.150258 2	0	0	74
Sridhar Chilimuri et al, 2020	Cohort	91%	Safety Net Hospital - Bronx, New York	375	93	0	0	0	246	0	36	N/A	63	Hospital	24	NY-13	46298	7.5	1185	460	4210	1.5	N/A	N/A	63
Mark W. Tenforde et al, 2020	Cross-Sectional	100%	11 Academic Medical Centers in 9 states	350	73	116	0	0	116	0	42	N/A	43	Hospital	N/A	N/A	N/A	N/A	N/A	N/A	N/A	N/A	N/A	N/A	47
Jeremy A W Gold et al, 2020	Cross-Sectional	75%	8 Hospitals in Georgia	297	247	50	0	0	0	0	0	N/A	N/A	Hospital	27	GA-5	60247	16.6	1873.3	2210	7500	0.5	N/A	38	N/A
Fahad Marmarchi et al, 2020	Cohort	82%	Teaching Hospital in Atlanta - Georgia	288	209	0	0	0	0	0	79	63	N/A	Hospital	N/A	N/A	N/A	14	900	410	4273	2.079961 7	N/A	N/A	55

© 2021 Magesh S et al. *JAMA Network Open.*

eTable 2: Study Summary Characteristics for Comorbidities

Summary characteristics for each of the 68 studies included in this systematic review and meta-analysis, including but not limited to the following variables: percent of ever smokers, median BMI, BMI over 40, cardiovascular disease, hypertension, COPD, diabetes, and malignancy/cancer.

Study First Author	Percent of Ever Smokers	Median BMI	Percent BMI Over 40	Percent Cardiovascular Disease	Percent Hypertension	Percent COPD	Percent Diabetes	Percent Malignancy/Cancer
David P. Bui et al, 2020	N/A	N/A	N/A	N/A	N/A	N/A	N/A	N/A
Di Xiong et al, 2020	N/A	N/A	N/A	N/A	N/A	N/A	N/A	N/A
Erin K. Stokes et al, 2020	N/A	N/A	N/A	32.2	N/A	N/A	30.2	N/A
Ishaan Pathak et al, 2020	N/A	N/A	N/A	N/A	N/A	N/A	N/A	N/A
Mary L. Adams et al, 2020	N/A	N/A	N/A	N/A	N/A	N/A	N/A	N/A
Michael Poulson et al, 2020	N/A	N/A	N/A	N/A	N/A	N/A	N/A	N/A
Sara J. Cromer et al, 2020	N/A	N/A	N/A	N/A	N/A	N/A	N/A	N/A
Jon Zelner et al, 2020	N/A	N/A	N/A	N/A	N/A	N/A	N/A	N/A
Diego A. Martinez et al, 2020	N/A	N/A	N/A	N/A	N/A	N/A	N/A	N/A
Yelena Rozenfeld et al, 2020	N/A	N/A	N/A	N/A	N/A	N/A	14.3	N/A

© 2021 Magesh S et al. *JAMA Network Open.*

Jacob McPadden et al, 2020	N/A	N/A	N/A	N/A	53	N/A	32.5	5.4
Samuel B Reichberg et al, 2020	N/A	N/A	N/A	N/A	N/A	N/A	N/A	N/A
Farhaan S Vahidy et al, 2020	N/A	N/A	N/A	N/A	47.2	N/A	24.2	N/A
Sharia M Ahmed et al, 2020	N/A	N/A	N/A	N/A	14.53	N/A	N/A	N/A
Kristen M. J. Azar et al, 2020	27.4	N/A	N/A	10.2	29.8	9.2	N/A	6.8
Alan Pan et al, 2020	17.511	N/A	N/A	N/A	N/A	12.5	0	0
Lara Jehi et al, 2020	N/A	N/A	N/A	N/A	N/A	N/A	N/A	N/A
Baligh R Yehia et al, 2020	N/A	N/A	N/A	19.99	23.2	8.47	2585	2.71
George N. Ioannou et al, 2020	N/A	N/A	N/A	N/A	62.1	18.8	38.1	22.7
Gbenga Ogedegbe et al, 2020	24.7	28.2	51.4	N/A	N/A	N/A	N/A	N/A
Rafi Kabarriti et al, 2020	N/A	N/A	N/A	22.1	44.7	N/A	32.9	5.6
Michael Gottlieb et al, 2020	14	27.2	264	N/A	22.1	1.3	14.6	6.2
Brian E. Dixon et al, 2020	N/A	N/A	N/A	N/A	N/A	N/A	N/A	N/A

© 2021 Magesh S et al. *JAMA Network Open.*

Haotian Chen et al, 2020	22	N/A	N/A	N/A	21.99	N/A	N/A	N/A
Fatima Rodriguez et al, 2020	6.6	N/A	N/A	9.5	60.1	18.7	37	11.2
Ahmad Khan et al, 2020	N/A	N/A	N/A	N/A	36.057	21.5	19.1	N/A
Sara Y. Tartof et al, 2020	21.24	N/A	9.16	3.26	24.47	12.56	20.12	2.22
Michelle A Waltenburg et al, 2020	N/A	N/A	N/A	N/A	N/A	N/A	N/A	N/A
Tian Gu et al, 2020	33.5	N/A	N/A	N/A	N/A	N/A	N/A	N/A
Nicholas E. Ingraham et al, 2020	N/A	N/A	N/A	N/A	66.3	14.2	N/A	N/A
S.B. Chan et al, 2020	N/A	N/A	N/A	N/A	N/A	N/A	N/A	N/A
Kengo Inagaki et al, 2020	N/A	N/A	N/A	N/A	N/A	N/A	N/A	N/A
Ayodeji Adegunsoye et al, 2020	N/A	N/A	N/A	N/A	N/A	N/A	N/A	N/A
Nir Menachemi et al, 2020	N/A	N/A	N/A	N/A	N/A	N/A	N/A	N/A
Eboni G Price-Haywood et al, 2020	N/A	N/A	N/A	3.99	30.8	2.26	16.25	4.5

© 2021 Magesh S et al. *JAMA Network Open.*

Heather E. Hsu et al, 2020	N/A	N/A	N/A	14.9	45.7	5.3	25.9	7.1
Amy K Feehan et al, 2020	N/A	N/A	N/A	N/A	N/A	N/A	N/A	N/A
L. Silvia Muñoz-Price et al, 2020	33.6	N/A	N/A	N/A	N/A	N/A	N/A	N/A
Rolando G. Valenzuela et al, 2020	N/A	N/A	N/A	8.3	27.5	4.7	14.38	N/A
Naima T. Joseph et al, 2020	N/A	N/A	N/A	N/A	N/A	N/A	N/A	N/A
Olga Grechukhina et al, 2020	0	0	0	0	0	0	0	0
Ana A. Weil et al, 2020	N/A	N/A	N/A	N/A	N/A	N/A	N/A	N/A
Jennifer Woo Baidal et al, 2020	6.94	N/A	N/A	71.6	N/A	N/A	N/A	11.5
Beth L. Pineles et al, 2020	N/A	N/A	N/A	N/A	23	N/A	10	N/A
Ingrid V Bassett et al, 2020	38	29.1	N/A	79	52	31	N/A	N/A
Brian T. Garibaldi et al, 2020	33	29	N/A	15	47	19	30	10
Benjamin D. Renelus et al, 2020	N/A	N/A	N/A	N/A	67.3	N/A	43.46	3.67

© 2021 Magesh S et al. *JAMA Network Open.*

Angelico Mendy et al, 2020	N/A	N/A	N/A	N/A	N/A	N/A	N/A	N/A
Nana-Yaa Misa et al, 2020	N/A	N/A	N/A	N/A	N/A	N/A	N/A	N/A
Priyank Shah et al, 2020	17	N/A	N/A	N/A	79.7	9	42.3	9.2
Wesley H Self et al, 2020	N/A	N/A	N/A	N/A	52.8	8.14	34.6	N/A
Sindhura Bandi et al, 2020	N/A	N/A	N/A	N/A	N/A	N/A	N/A	N/A
Ashish Bhargava et al, 2020	N/A	N/A	N/A	N/A	N/A	N/A	40.3	5.4
Vijay Gayam et al, 2020	N/A	N/A	N/A	N/A	N/A	N/A	N/A	N/A
Samina Bhumbra et al, 2020	N/A	N/A	N/A	N/A	N/A	N/A	N/A	N/A
Sridhar Chilimuri et al, 2020	N/A	N/A	N/A	17	60	N/A	N/A	N/A
Mark W. Tenforde et al, 2020	31	N/A	N/A	30	29	5	15	N/A
Jeremy A W Gold et al, 2020	N/A	N/A	12.7	25.6	67.5	5.2	39.7	3.9
Fahad Marmarchi et al, 2020	N/A	32	N/A	14	74	7	45	N/A

© 2021 Magesh S et al. *JAMA Network Open.*

Victoria Silver et al, 2020	38	N/A	23	32	80	13	52	12
Cian P. McCarthy et al, 2020	32.8	28.5	N/A	49	51.8	8.9	27.5	27.5
James Andrew McCracken et al, 2020	9.5	29	N/A	24.5	84.5	8.5	57.5	6
Ilona Telefus Goldfarb et al, 2020	N/A	N/A	N/A	N/A	N/A	N/A	8.19	N/A
Christopher S. King et al, 2020	N/A	30	N/A	6.7	52	N/A	34.1	N/A
Vikramjit Mukherjee et al, 2020	28.5	N/A	9.7	13.9	51.1	2.9	37.2	2.9
Mark P. Abrams et al, 2020	N/A	27.2	N/A	77.4	82.7	16.5	52.6	N/A
Stephen Capone et al, 2020	N/A	N/A	N/A	N/A	59.8	N/A	49	N/A
Anthony M. Valeri et al, 2020	32	25.2	N/A	46	98	17	69	N/A

© 2021 Magesh S et al. *JAMA Network Open.*

eTable 3. Adjustment of Relative Risk Ratios (RRs) for Additional Variables

The following variables were adjusted for RR for each racial/ethnic group by COVID-19 outcome: age, ADI, county median income, a combined measure of clinical care quality, urban core opportunity index, and a combined measure of medical comorbidities.

		Age-Adjusted		ADI-Adjusted		Income-Adjusted		Clinical Care-Adjusted (combined)			UOI		Comorbidities		
	**# of studies**	**RR (95% CI)**	**RR *p*-value**	**RR (95% CI)**	**RR *p*-value**	**RR (95% CI)**	**RR *p*-value**	**RR (95% CI)**	**RR *p*-value**	***alpha***	**RR (95% CI)**	**RR *p*-value**	**RR (95% CI)**	**RR *p*-value**	***alpha***
**Cohort**															
**(1) Positive**															
White	13	Reference	N/A	Reference	N/A	Reference	N/A	Reference	N/A		Reference	N/A	Reference	N/A	
African American	20	1.34 (.91, 1.97)	0.137	2.01 (1.04, 3.88)	0.037	1.92 (1.00, 3.66)	0.048	1.79 (1.11, 3.17)	0.029	0.902	6.12 (0.11, 337.85)	0.383	3.34 (0.50, 23.59)	0.212	0.958
Hispanic and Latino	10	6.98 (2.06, 23.58)	0.002	2.09 (1.13, 3.88)	0.019	3.26 (1.50, 7.07)	0.003	N/A	N/A	0.088	2.15 (0.98, 4.74)	0.056	1.98 (1.30, 3.02)	0.002	N/A
Asian-American	7	N/A	N/A	1.12 (1.04, 1.21)	0.003	1.14 (1.05, 1.25)	0.003	1.116 (1.03, 1.31)	0.015	0.890	1.13 (1.07, 1.19)	<.001	N/A	N/A	N/A
**(2) Hospitalization**															
White	4	Reference	N/A	Reference	N/A	Reference	N/A	Reference	N/A	N/A	Reference	N/A	Reference	N/A	
African American	4	N/A	N/A	N/A	N/A	N/A	N/A	N/A	N/A	N/A	N/A	N/A	N/A	N/A	N/A
Hispanic and Latino	3	N/A	N/A	N/A	N/A	N/A	N/A	N/A	N/A	N/A	N/A	N/A	N/A	N/A	N/A
Asian-American	0	N/A	N/A	N/A	N/A	N/A	N/A	N/A	N/A	N/A	N/A	N/A	N/A	N/A	N/A
**(3) ICU**															
White	4	Reference	N/A	Reference	N/A	Reference	N/A	Reference	N/A	N/A	Reference	N/A	Reference	N/A	
African American	4	N/A	N/A	N/A	N/A	1.07 (0.63, 1.84)	0.816	N/A	N/A	N/A	1.11 (0.66, 1.87)	0.707	N/A	N/A	N/A
Hispanic and Latino	4	N/A	N/A	N/A	N/A	N/A	N/A	N/A	N/A	N/A	1.29 (0.66, 2.52)	0.465	N/A	N/A	N/A
Asian-American	3	.33 (0.07, 1.58)	0.164	N/A	N/A	N/A	N/A	N/A	N/A	N/A	N/A	N/A	N/A	N/A	N/A
**(4) Mortality**															
White	6	Reference	N/A	Reference	N/A	Reference	N/A	Reference	N/A	N/A	Reference	N/A	Reference	N/A	
African American	7	N/A	N/A	0.84 (0.62, 1.13)	0.258	0.85 (0.82, 0.88)	<0.001	N/A	N/A	N/A	N/A	N/A	N/A	N/A	N/A
Hispanic and Latino	5	N/A	N/A	N/A	N/A	0.59 (0.26, 1.34)	0.209	N/A	N/A	N/A	N/A	N/A	N/A	N/A	N/A
Asian-American	4	N/A	N/A	1.18 (0.99, 1.41)	0.066	1.18 (0.69, 2.00)	0.553	N/A	N/A	N/A	N/A	N/A	N/A	N/A	N/A
**Cross-Sectional**															

© 2021 Magesh S et al. *JAMA Network Open.*

**(4) Mortality**															
White	5	Reference	N/A	Reference	N/A	Reference	N/A	Reference	N/A		Reference	N/A	Reference	N/A	N/A
African American	6	0.92 (0.70, 1.20)	0.555	0.88 (0.63, 1.22)	0.457	0.91 (0.68, 1.23)	0.554	0.99 (0.91, 1.07)	0.819	0.779	N/A	N/A	0.86 (0.62, 1.19)	0.370	0.866
Hispanic and Latino	4	N/A	N/A	0.44 (0.31, 0.61)	<0.001	0.43 (0.41, 0.46)	<0.001	N/A	N/A	N/A	N/A	N/A	N/A	N/A	N/A
Asian-American	4	N/A	N/A	0.73 (0.32, 1.68)	0.465	0.44 (0.36, 0.54)	<0.001	0.74 (0.59, 0.94)	0.011	0.874	N/A	N/A	N/A	N/A	N/A

© 2021 Magesh S et al. *JAMA Network Open.*

eTable 4. Adjustment of Odds Ratios (ORs) for Additional Variables

The following variables were adjusted for OR for each racial/ethnic group by COVID-19 outcome: age, ADI, county median income, a combined measure of clinical care quality, urban core opportunity index, and a combined measure of medical comorbidities.

		Age-Adjusted		ADI-Adjusted		Income-Adjusted		Clinical Care-Adjusted (combined)			UOI		Comorbidites		
	**# of studies**	**OR (95% CI)**	**OR p-value**	**OR (95% CI)**	**OR p-value**	**OR (95% CI)**	**OR p-value**	**OR (95% CI)**	**OR p-value**	***alpha***	**OR (95% CI)**	**OR p-value**	**OR (95% CI)**	**OR p-value**	***alpha***
**Cohort**															
**(1) Positive**															
White	13	Reference	N/A	Reference	N/A	Reference	N/A	Reference	N/A		Reference	N/A	Reference	N/A	
African American	20	5.02 (1.57, 16.06)	0.007	2.69 (0.99, 7.32)	0.052	2.55 (0.92, 7.04)	0.071	2.45 (1.67, 3.58)	<0.001	0.902	N/A	N/A	6.20 (0.34, 114.5)	0.220	0.958
Hispanic and Latino	10	4.38 (1.22, 15.73)	0.233	2.52 (1.20, 5.28)	0.014	4.15 (1.70, 10.13)	0.002	N/A	N/A	0.089	2.71 (1.37, 5.35)	0.004	2.33 (1.56, 3.47)	<0.001	N/A
Asian-American	7	N/A	N/A	1.31 (1.12, 1.53)	0.221	1.34 (1.13, 1.61)	0.001	1.35 (1.06, 1.72)	0.015	0.890	1.31 (1.19, 1.45)	<0.001	N/A	N/A	N/A
**(2) Hospitalization**															
White	4	Reference	N/A	Reference	N/A	Reference	N/A	Reference	N/A	N/A	Reference	N/A	Reference	N/A	
African American	4	N/A	N/A	1.94 (0.07, 53.28)	0.713	N/A	N/A	N/A	N/A	N/A	N/A	N/A	N/A	N/A	N/A
Hispanic and Latino	3	N/A	N/A	N/A	N/A	N/A	N/A	N/A	N/A	N/A	N/A	N/A	N/A	N/A	N/A
Asian-American	0	N/A	N/A	N/A	N/A	N/A	N/A	N/A	N/A	N/A	N/A	N/A	N/A	N/A	N/A
**(3) ICU**															
White	4	Reference	N/A	Reference	N/A	Reference	N/A	Reference	N/A	N/A	Reference	N/A	Reference	N/A	
African American	4	N/A	N/A	N/A	N/A	1.11 (0.54, 2.25)	0.787	N/A	N/A	N/A	1.16 (0.59, 2.27)	0.679	N/A	N/A	N/A
Hispanic and Latino	4	N/A	N/A	N/A	N/A	1.41 (0.58, 3.41)	0.455	N/A	N/A	N/A	1.43 (0.57, 3.59))	0.454	N/A	N/A	N/A
Asian-American	3	2.09 (1.59, 2.76)	<0.001	N/A	N/A	0.87 (0.63, 1.18)	0.391	N/A	N/A	N/A	N/A	N/A	N/A	N/A	N/A
**(4) Mortality**															
White	6	Reference	N/A	Reference	N/A	Reference	N/A	Reference	N/A	N/A	Reference	N/A	Reference	N/A	
African American	7	N/A	N/A	0.82 (0.56, 1.19)	0.306	0.84 (0.81, 0.87)	<0.001	N/A	N/A	N/A	N/A	N/A	N/A	N/A	N/A
Hispanic and Latino	5	N/A	N/A	0.88 (0.50, 1.53)	0.667	0.58 (0.24, 1.39)	0.226	N/A	N/A	N/A	N/A	N/A	N/A	N/A	N/A
Asian-American	4	N/A	N/A	1.35 (0.96, 1.89)	0.082	1.3 (0.30, 2.77)	0.656	N/A	N/A	N/A	N/A	N/A	N/A	N/A	N/A

© 2021 Magesh S et al. *JAMA Network Open.*

**Cross-Sectional**															
**(4) Mortality**															
White	5	Reference	N/A	Reference	N/A	Reference	N/A	Reference	N/A		Reference	N/A	Reference	N/A	N/A
African American	6	0.73 (0.39, 1.35)	0.325	0.86 (0.60, 1.25)	0.428	0.91 (0.67, 1.23)	0.055	0.98 (0.89, 1.08)	0.695	0.779	N/A	N/A	0.84 (0.56, 1.24)	0.859	0.866
Hispanic and Latino	4	N/A	N/A	0.42 (0.29, 0.61)	<0.001	0.42 (0.39, 0.44)	<0.001	N/A	N/A	N/A	N/A	N/A	N/A	N/A	N/A
Asian-American	4	N/A	N/A	0.58 (0.16, 2.04)	0.409	0.39 (0.31, 0.48)	<0.001	0.62 (0.29, 1.33)	0.220	0.874	N/A	N/A	N/A	N/A	N/A

© 2021 Magesh S et al. *JAMA Network Open.*

eTable 5. Combined Prevalence of Cohort and Cross-sectional Studies

Combined prevalence of COVID-19 positivity, mortality, ICU admission, and hospitalization in cohort and cross-sectional studies.

	# of studies	Prevalence (per 1000 people)	I^2 (%)
**Cohort**			
(1) Positive			
White	13	296.58	100
African American	20	306.04	100
Hispanic and Latino	10	370.67	99
Asian-American	7	49.42	98
(2) Hospitalization			
White	4	98.69	100
African American	4	151.37	100
Hispanic and Latino	3	100.88	100
Asian-American	0	N/A	N/A
(3) ICU			
White	4	235.75	92
African American	4	243.52	97
Hispanic and Latino	4	288.65	98
Asian-American	3	409.26	85
(4) Mortality			
White	6	161.12	99
African American	7	143.99	99
Hispanic and Latino	5	130.51	100
Asian-American	4	42.99	98
**Cross-Sectional**			
(4) Mortality			
White	5	83.78	100
African American	6	89.37	99
Hispanic and Latino	4	26.73	88
Asian-American	4	63.54	93

© 2021 Magesh S et al. *JAMA Network Open.*

eTable 6. Summary of Q and I^2 Statistics for Study Variables

Q and I^2 statistics for correlations between proportions of individuals from each racial/ethnic group by COVID-19 outcome and the following variables: ADI, county median income, and measures of clinical care quality.

Variable Studied	No. of Studies	Q Statistic	I^2 Statistic, %
**(1) Cohort Studies**			
Proportion of Deceased Whites with ADI	6/69	101.24	97.36
Proportion of Deceased African Americans with ADI	6/69	943.43	99.62
Proportion of Deceased Hispanics/Latinos with ADI	4/69	115.03	97.4
Proportion of Deceased Asian-Americans with ADI	4/69	111.85	98.59
Proportion of Deceased Whites with county median income	6/69	250.99	98.68
Proportion of Deceased African Americans with county median income	6/69	324.41	99.2
Proportion of Deceased Hispanics/Latinos with county median income	4/69	204.09	98.55
Proportion of Deceased Asian-Americans with county median income	4/69	13.43	85.3
Proportion of Deceased African Americans with Preventable Hospital Stay	3/69	124.91	99.2
Proportion of Deceased African Americans with Primary Care Availability	3/69	127.84	99.22
Proportion of Deceased African Americans with Uninsured	3/69	119.2	99.16
Proportion of ICU Admitted Whites with county median income	3/69	1.33	25.06
Proportion of ICU Admitted African Americans with county median income	3/69	13.61	92.66
Proportion of ICU Admitted Hispanics/Latinos with county median income	3/69	57.62	98.26
Proportion of COVID-19 Positive Whites with ADI	6/69	402.13	99.07

© 2021 Magesh S et al. *JAMA Network Open.*

Proportion of COVID-19 Positive African Americans with ADI	13/69	14005.48	99.91
Proportion of COVID-19 Positive Hispanics/Latinos with ADI	6/69	238.09	99.72
Proportion of COVID-19 Positive Asian-Americans with ADI	4/69	197.72	98.63
Proportion of COVID-19 Positive Whites with county median income	9/69	2657.67	99.77
Proportion of COVID-19 Positive African Americans with county median income	15/69	4956.89	99.87
Proportion of COVID-19 Positive Hispanics/Latinos with county median income	9/69	497.21	99.06
Proportion of COVID-19 Positive Asian-Americans with county median income	5/69	395.16	99.25
Proportion of COVID-19 Positive Whites with Preventable Hospital Stay	7/69	2212.72	99.71
Proportion of COVID-19 Positive African Americans with Preventable Hospital Stay	9/69	2897.78	99.87
Proportion of COVID-19 Positive Hispanics/Latinos with Preventable Hospital Stay	5/69	201.58	99.69
Proportion of COVID-19 Positive Asian-Americans with Preventable Hospital Stay	4/69	34.15	93.3
Proportion of COVID-19 Positive Whites with Primary Care Availability	7/69	1999.8	99.73
Proportion of COVID-19 Positive African Americans with Primary Care Availability	9/69	3281.45	99.87
Proportion of COVID-19 Positive Hispanics/Latinos with Primary Care Availability	5/69	23.81	97.48
Proportion of COVID-19 Positive Asian-Americans with Primary Care Availability	4/69	12.22	81.45

© 2021 Magesh S et al. *JAMA Network Open.*

Proportion of COVID-19 Positive Whites with Uninsured	7/69	2028.71	99.67
Proportion of COVID-19 Positive African Americans with Uninsured	9/69	1561.64	99.74
Proportion of COVID-19 Positive Hispanics/Latinos with Uninsured	5/69	494.7	99.78
Proportion of COVID-19 Positive Asian-Americans with Uninsured	7/69	48.96	96.44
**(2) Cross-sectional**			
Proportion of Deceased Whites with ADI	6/69	101.24	97.36
Proportion of Deceased African Americans with ADI	6/69	943.43	99.62
Proportion of Deceased Hispanics/Latinos with ADI	4/69	115.03	97.4
Proportion of Deceased Asian-Americans with ADI	4/69	111.85	98.59
Proportion of Deceased Whites with county median income	6/69	250.99	98.68
Proportion of Deceased African Americans with county median income	6/69	324.41	99.2
Proportion of Deceased Hispanics/Latinos with county median income	4/69	204.09	98.55
Proportion of Deceased Asian-Americans with county median income	4/69	13.43	85.3
Proportion of Deceased African Americans with Preventable Hospital Stay	3/69	124.91	99.2
Proportion of Deceased African Americans with Primary Care Availability	3/69	127.84	99.22
Proportion of Deceased African Americans with Uninsured	3/69	119.2	99.16
Proportion of ICU Admitted Whites with county median income	3/69	1.33	25.06
Proportion of ICU Admitted African Americans with county median income	3/69	13.61	92.66

© 2021 Magesh S et al. *JAMA Network Open.*

Proportion of ICU Admitted Hispanics/Latinos with county median income	3/69	57.62	98.26

© 2021 Magesh S et al. *JAMA Network Open.*

eFigure 1. PRISMA Workflow for Studies Included in Analysis

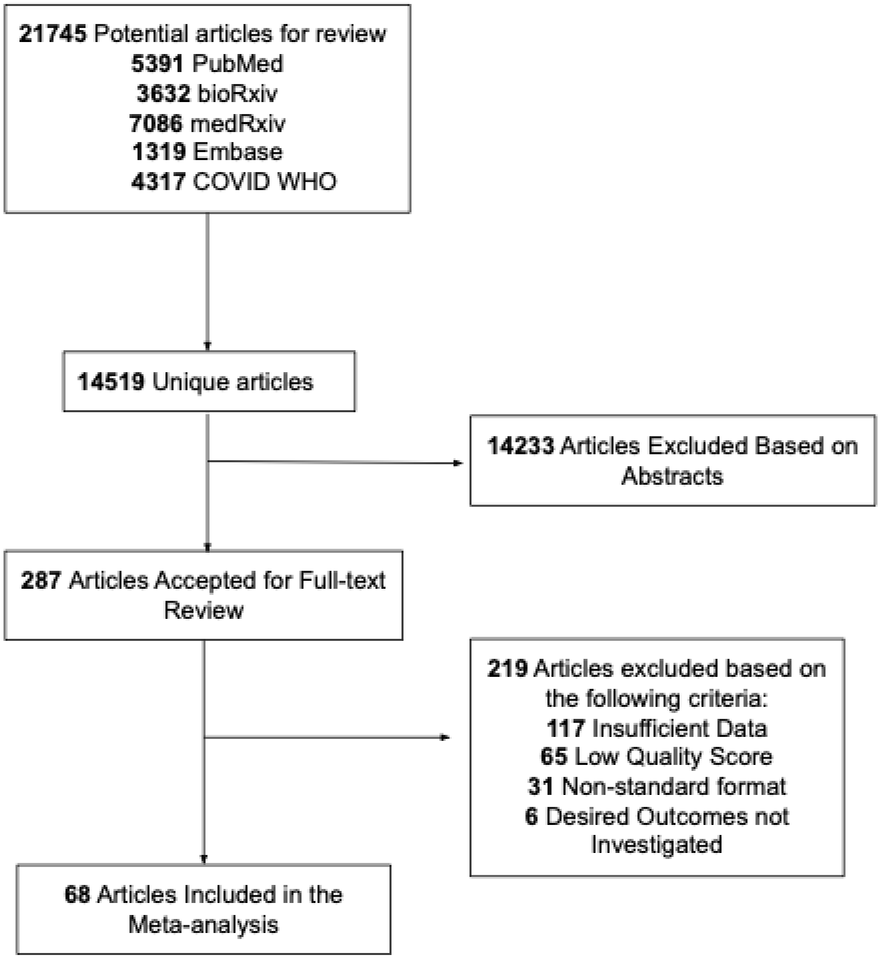

© 2021 Magesh S et al. *JAMA Network Open.*

eFigure 2. Funnel Plots for Deceased Individuals in Cohort and Cross-sectional Studies

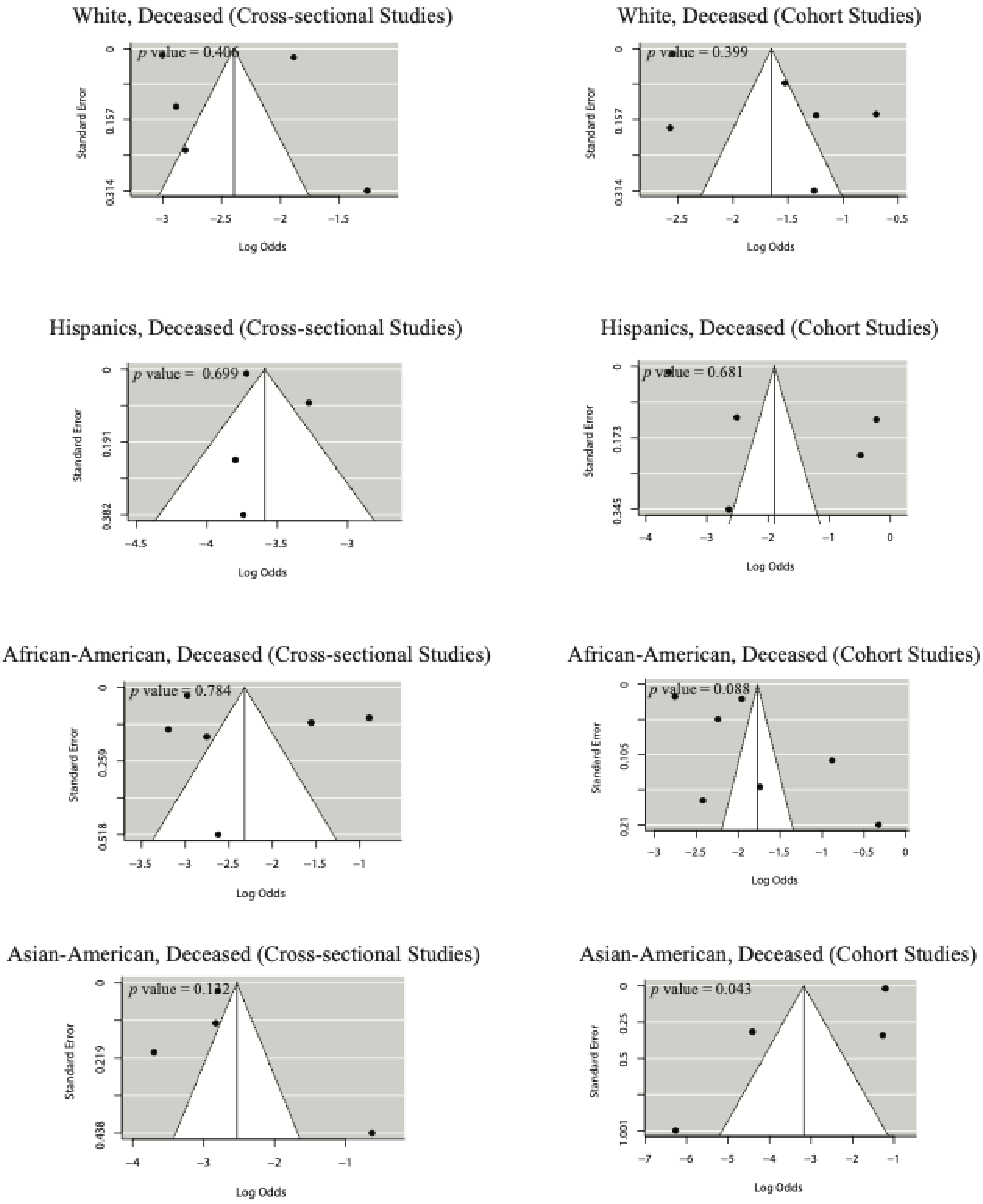

© 2021 Magesh S et al. *JAMA Network Open.*

eFigure 3. Funnel Plots for Patients Admitted to ICU or Hospitalized in Cohort Studies

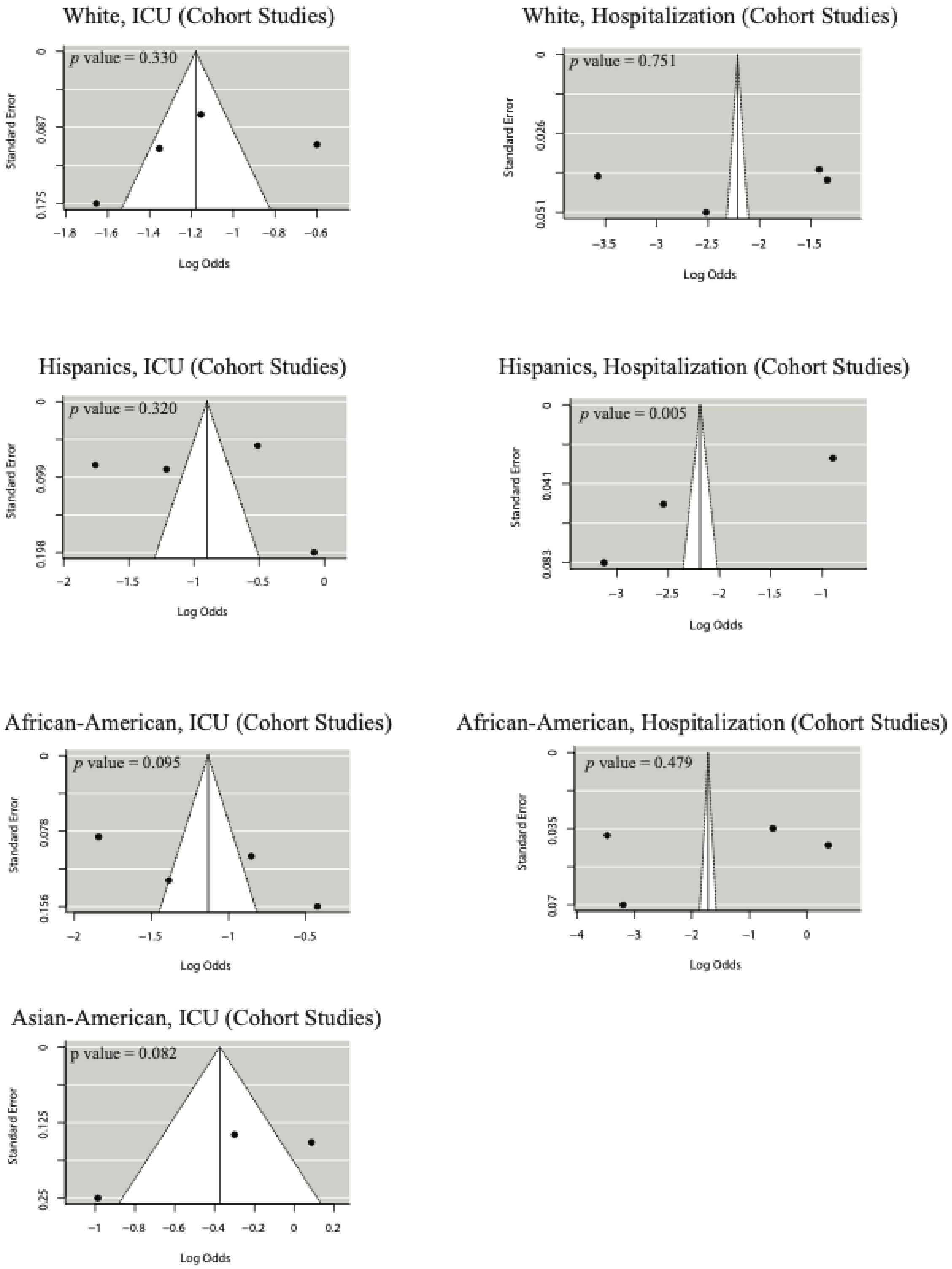

© 2021 Magesh S et al. *JAMA Network Open.*

eFigure 4. Funnel Plots for COVID-19 Positive Patients in Cohort and Cross-sectional Studies

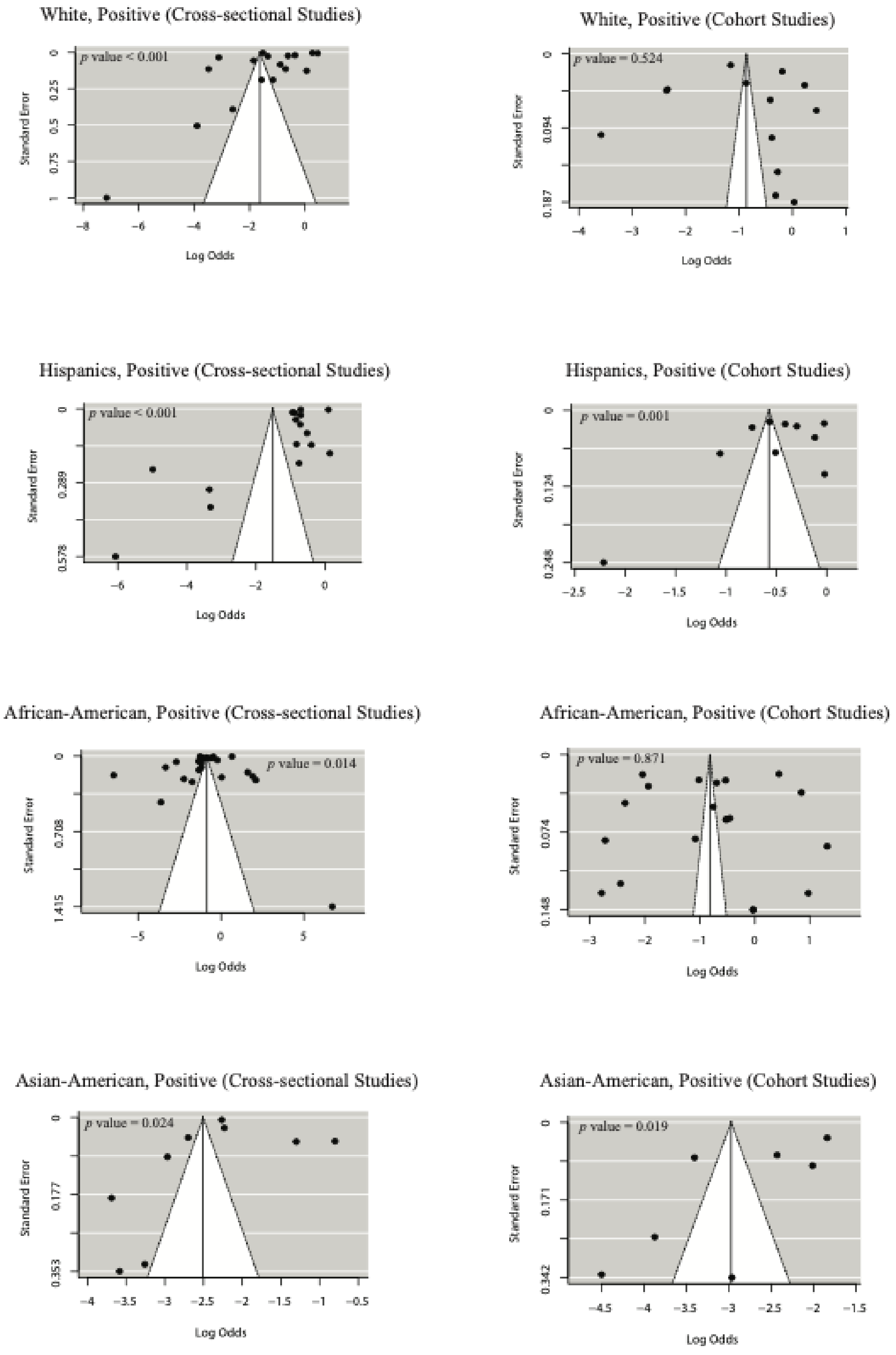

© 2021 Magesh S et al. *JAMA Network Open.*

eFigure 5. Forest Plot for COVID-19 Positivity in Cohort and Cross-sectional Studies

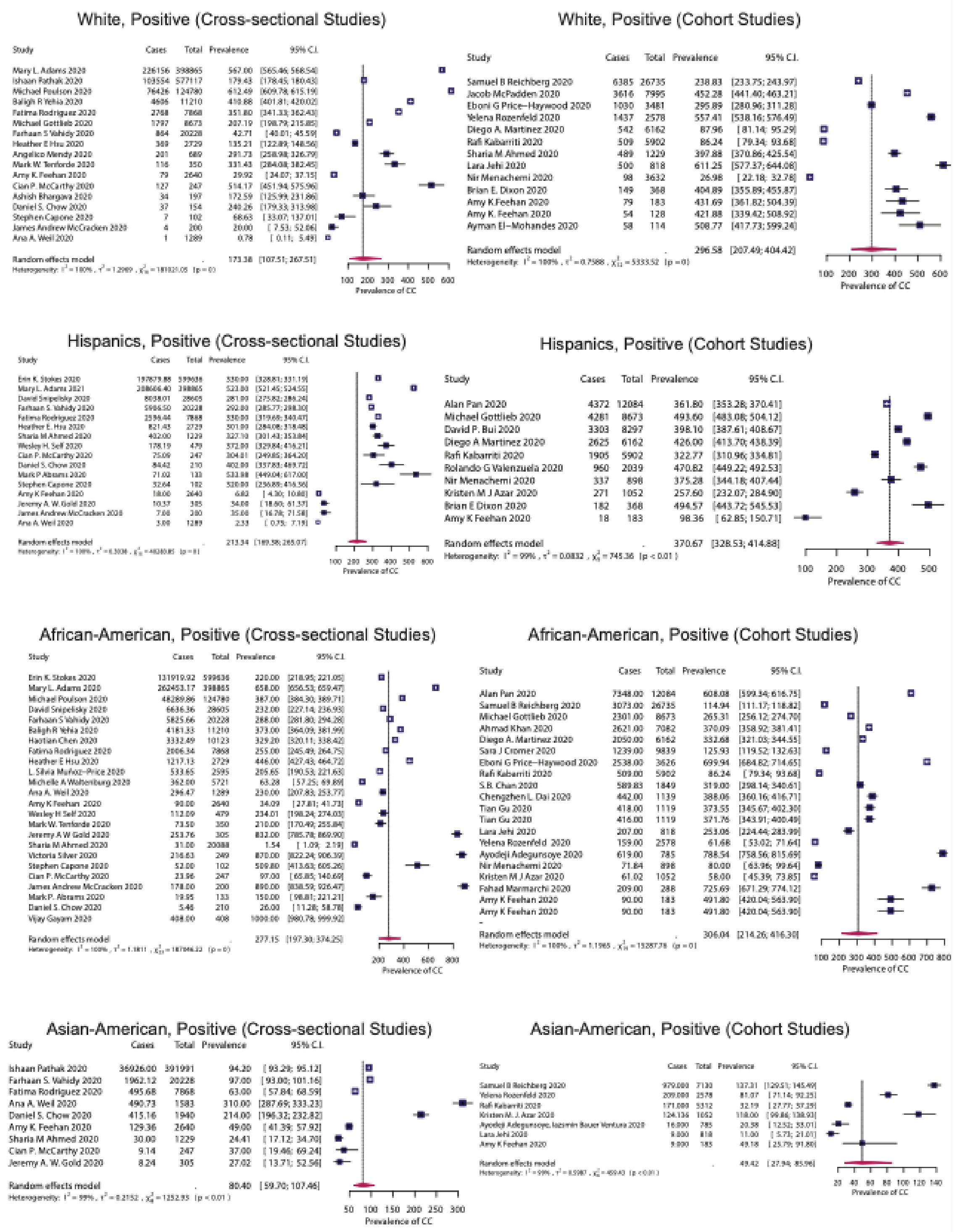

© 2021 Magesh S et al. *JAMA Network Open.*

eFigure 6. Forest Plot for Patients Admitted to ICU or Hospitalized in Cohort Studies

White, ICU (Cohort Studies)

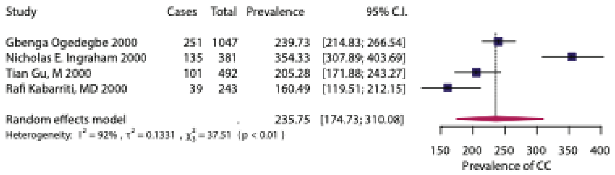

White, Hospitalization (Cohort Studies)

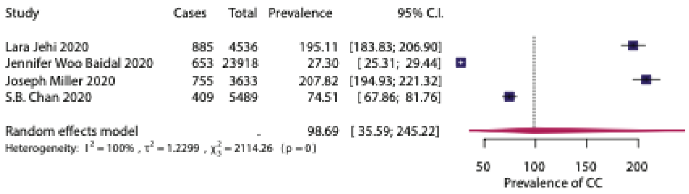

Hispanics, ICU (Cohort Studies)

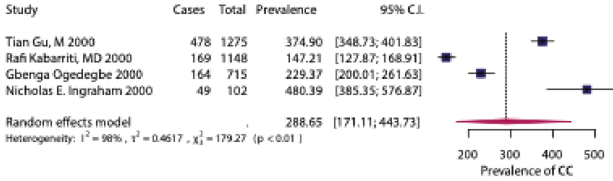

Hispanics, Hospitalization (Cohort Studies)

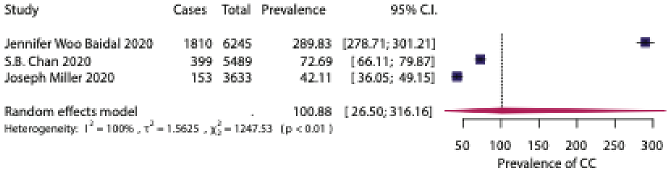

African-American, ICU (Cohort Studies)

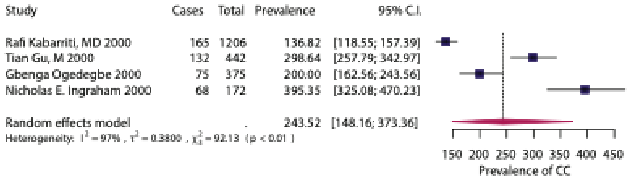

African-American, Hospitalization (Cohort Studies)

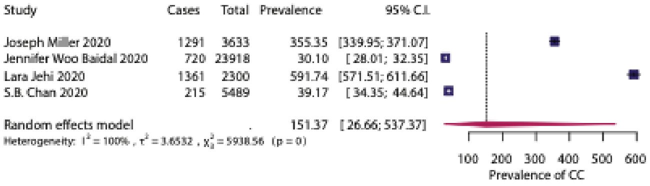

Asian-American, ICU (Cohort Studies)

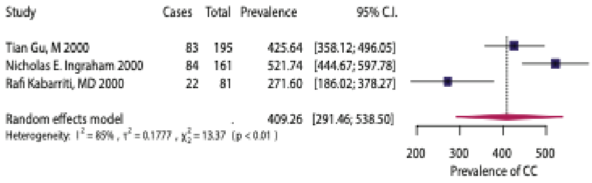

© 2021 Magesh S et al. *JAMA Network Open.*

eFigure 7. Forest Plot for Deceased Individuals in Cohort and Cross-sectional Studies

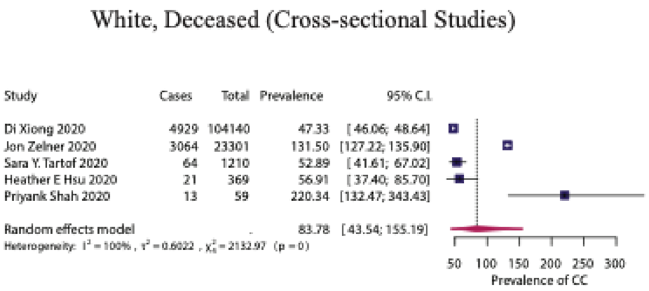

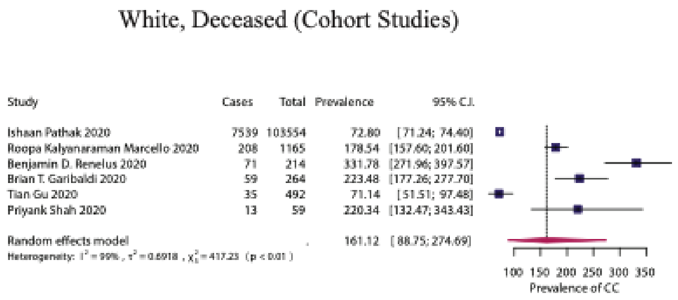

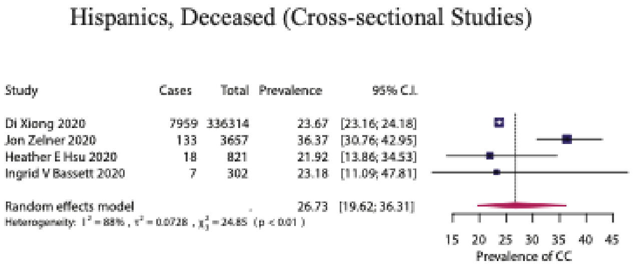

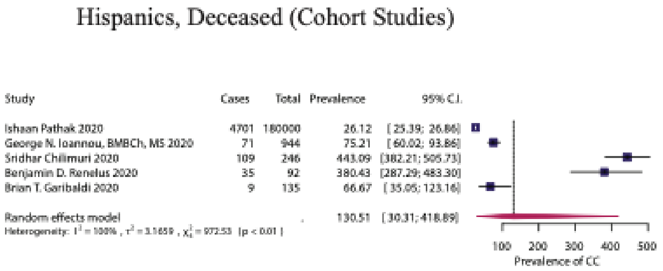

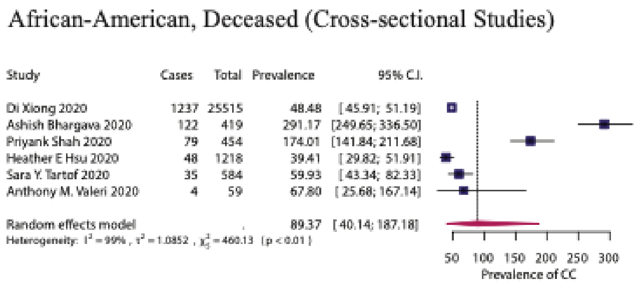

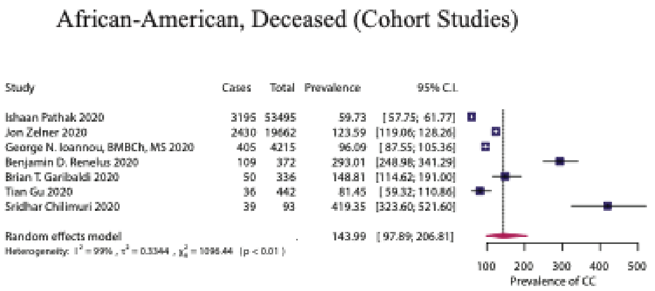

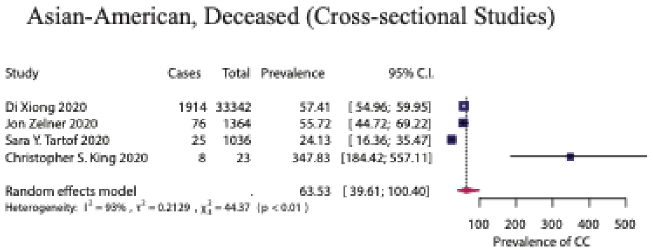

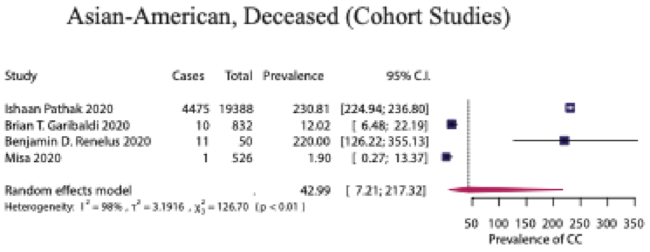

© 2021 Magesh S et al. *JAMA Network Open.*

eFigure 8. Metaregression for County Median Income

Meta-regression for county median income in (A) Asian Americans and Whites who are deceased (cohort and cross-sectional studies) and (B) Whites who were admitted to the ICU (cohort studies).

A

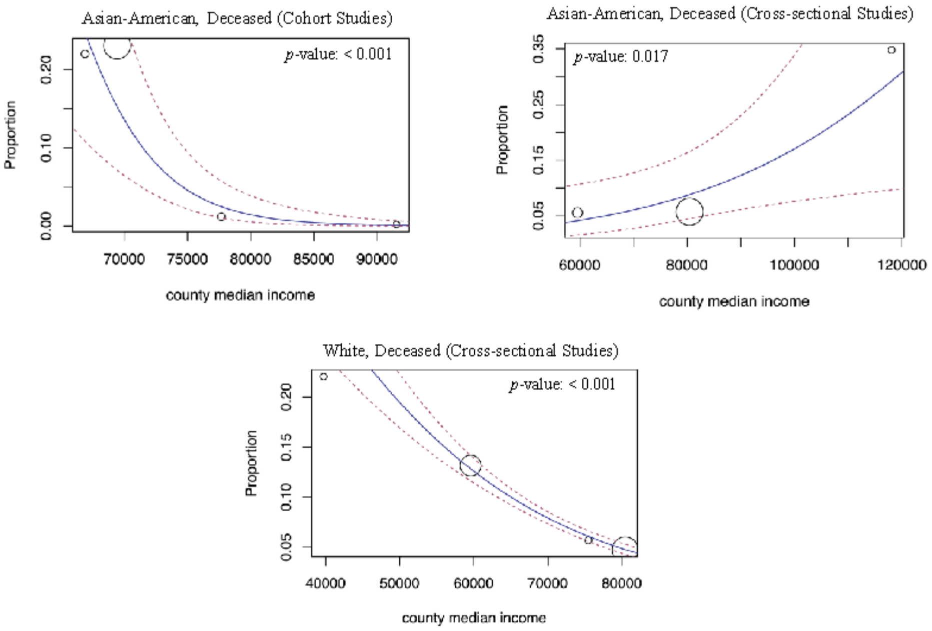

B

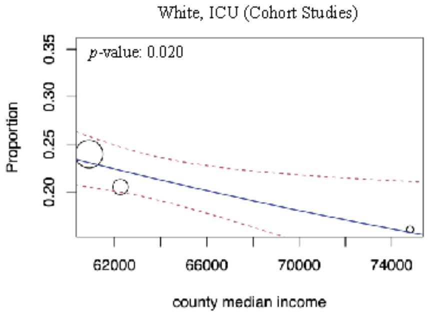

© 2021 Magesh S et al. *JAMA Network Open.*

eFigure 9. Spearman Correlations for Measures of Clinical Care Quality

Spearman Correlations for ADI and county median income in respect to the following variables: urban core opportunity index, population per one primary care physician, preventable hospital stays, and amount of uninsured individuals.

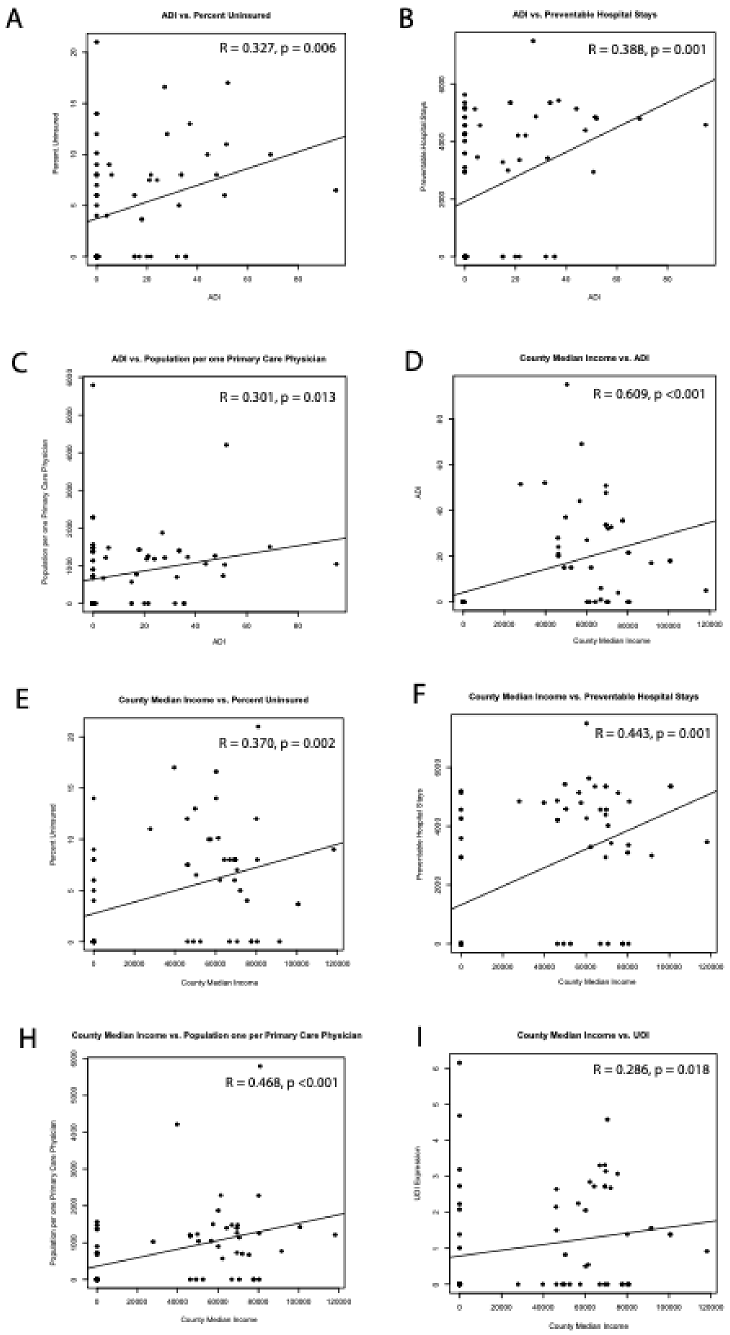

© 2021 Magesh S et al. *JAMA Network Open.*

eFigure 10. Metaregression for Clinical Care Measures

Meta-regression for measures of clinical care quality in the following cohorts: meta-regression for preventable hospital stays in correlation with Asian Americans who tested positive for COVID-19 in cohort studies; meta-regression for primary care physician availability in correlation with Asian Americans and Hispanics who tested positive for COVID-19 (cohort studies) and Whites who are deceased (cross-sectional studies); and meta-regression for the amount of uninsured individuals in correlation with African Americans who tested positive for COVID-19 (cohort studies) and Whites who are deceased (cross sectional studies).

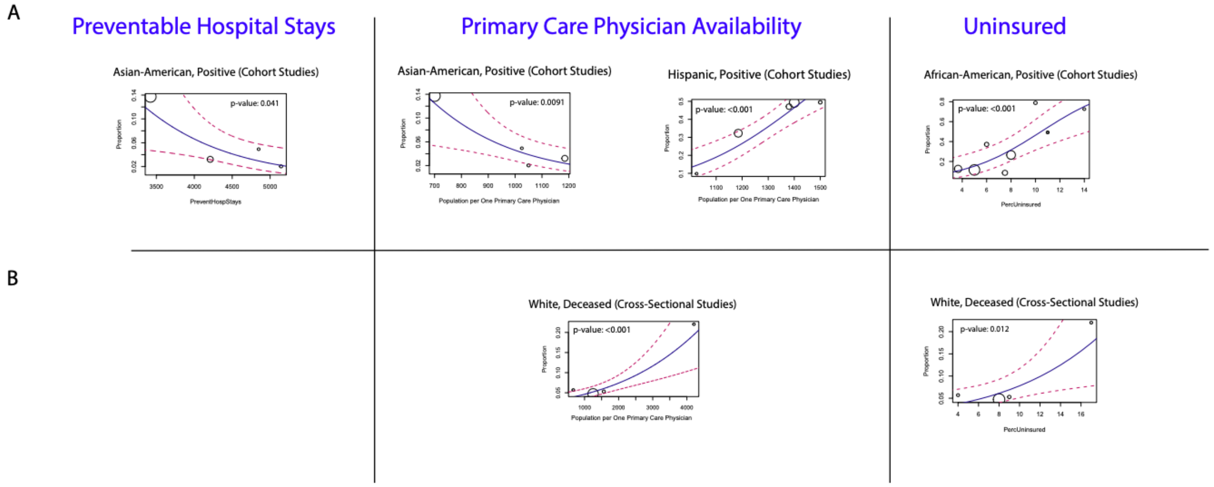

© 2021 Magesh S et al. *JAMA Network Open.*

eFigure 11. Leave-One-Out Sensitivity Analysis for Deceased Individuals in Cohort and Cross-sectional Studies

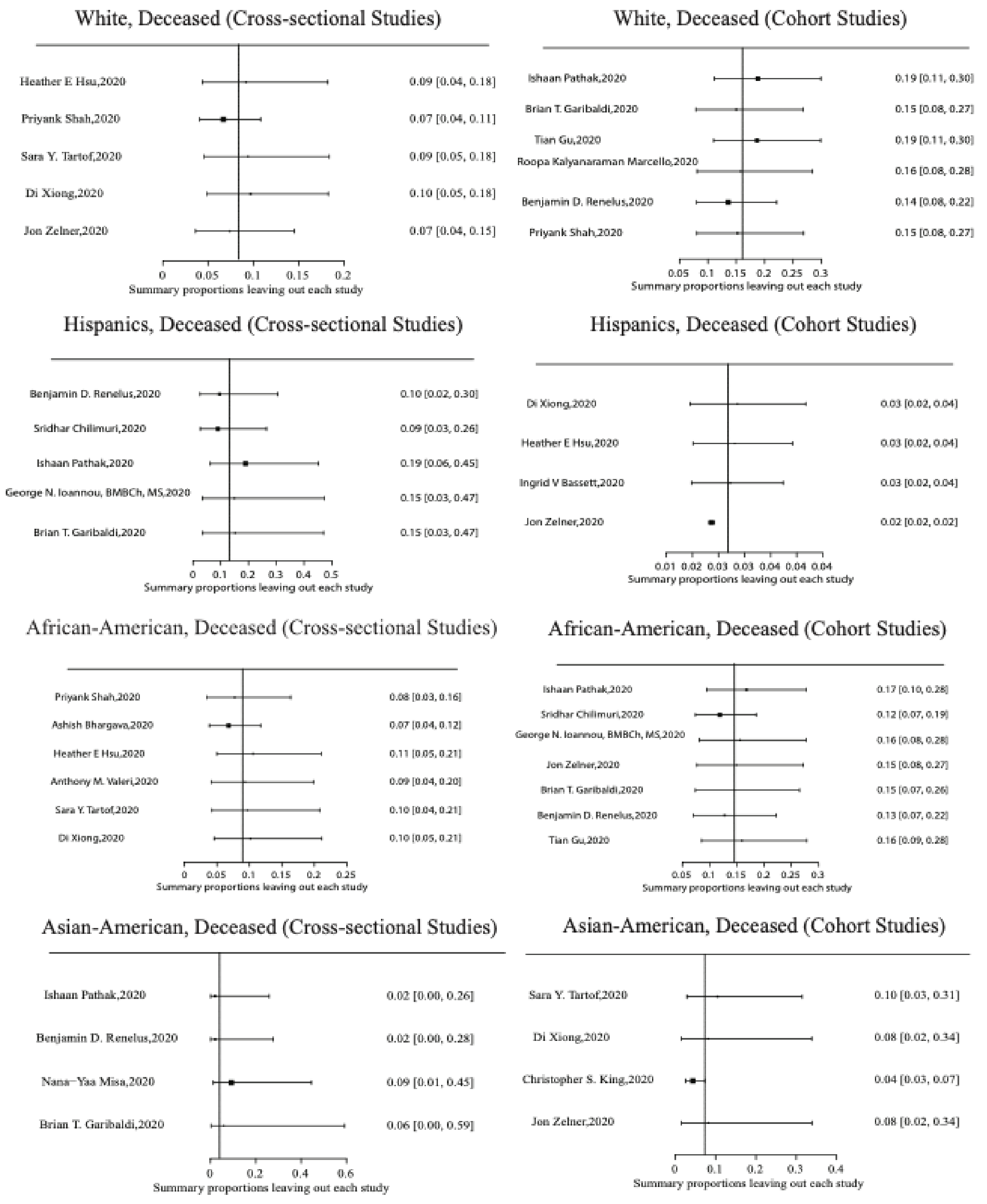

© 2021 Magesh S et al. *JAMA Network Open.*

eFigure 12. Leave-One-Out Sensitivity Analysis for Patients Admitted to ICU or Hospitalized in Cohort Studies

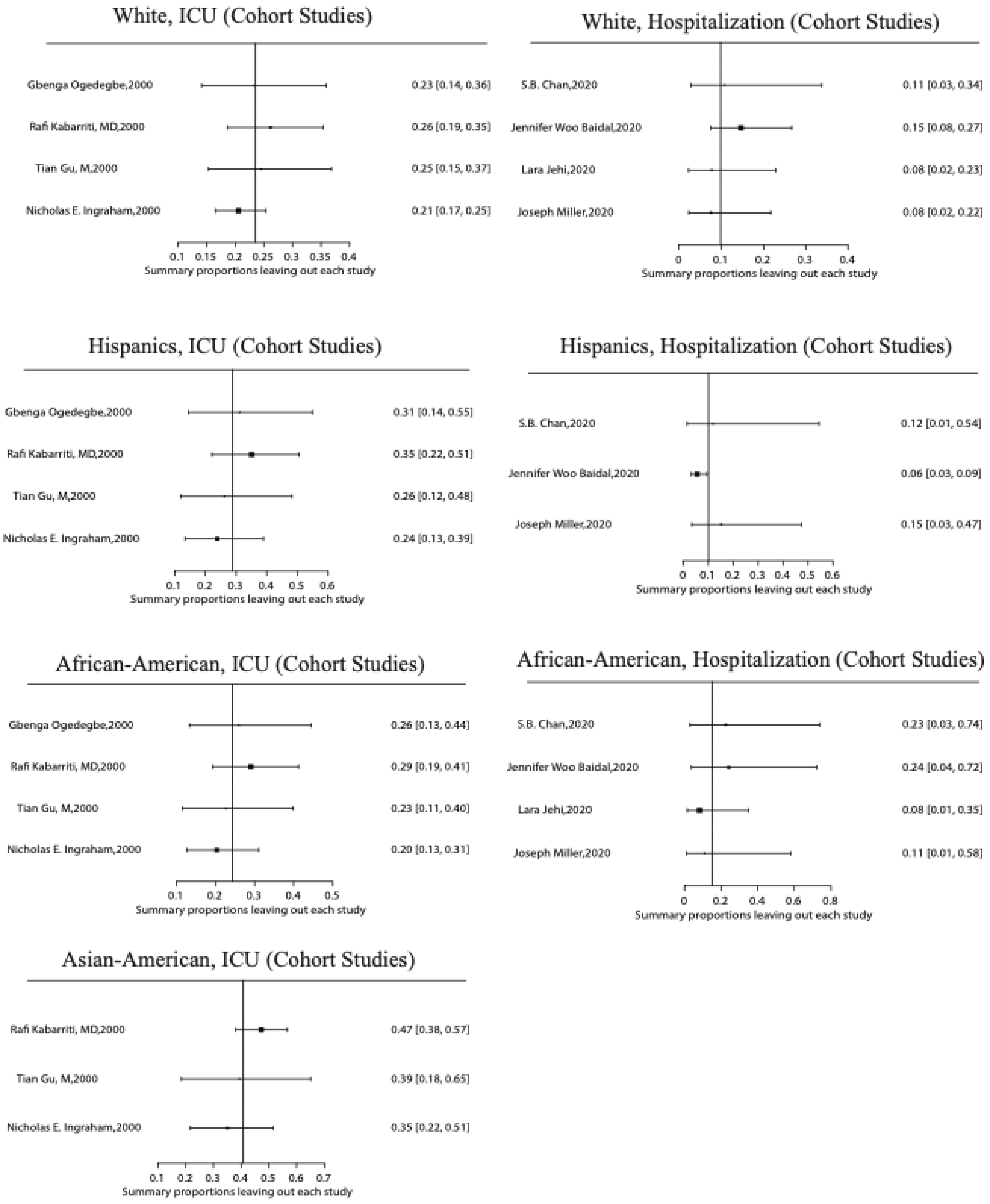

eFigure 13. Leave-One-Out Sensitivity Analysis for COVID-19 Positive Patients in Cohort and Cross-sectional Studies

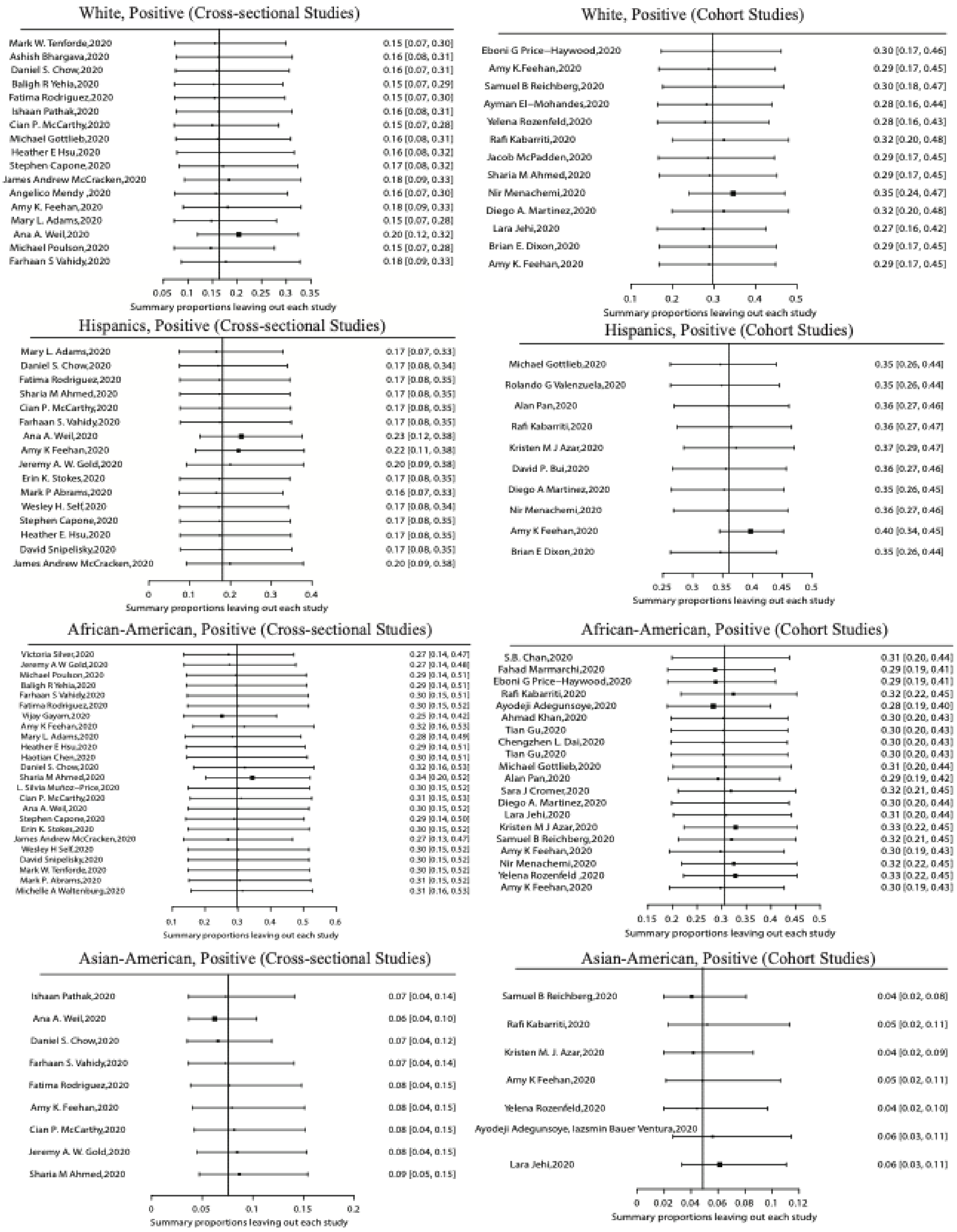

© 2021 Magesh S et al. *JAMA Network Open.*

eFigure 14. Forest Plots for Deceased Patients After Removing Dominating Studies

Adjusted forest plots for deceased patients following removal of outlier studies identified in leave-one-out sensitivity analysis.

White, Deceased (Cross-sectional Studies)

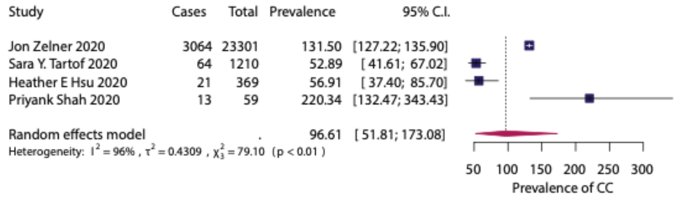

African-American, Deceased (Cross-sectional Studies)

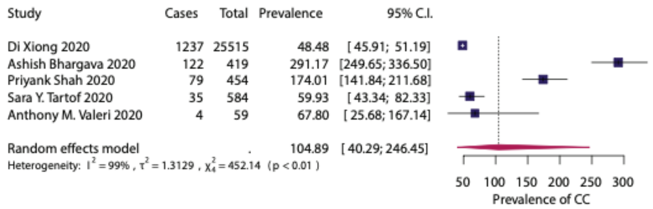

African-American, Deceased (Cohort Studies)

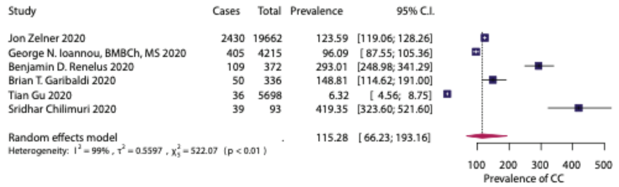

Asian-American, Deceased (Cross-sectional Studies)

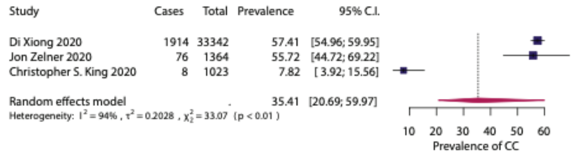

Asian-American, Deceased (Cohort Studies)

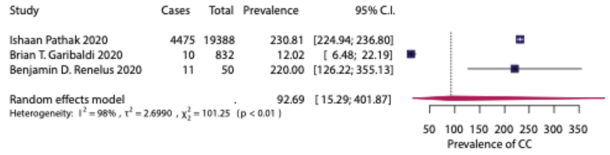

© 2021 Magesh S et al. *JAMA Network Open.*

eFigure 15. Forest Plots for Positive Individuals After Removing Dominating Studies
Adjusted forest plots for COVID-19 positive patients following removal of outlier studies identified in leave-one-out sensitivity analysis.

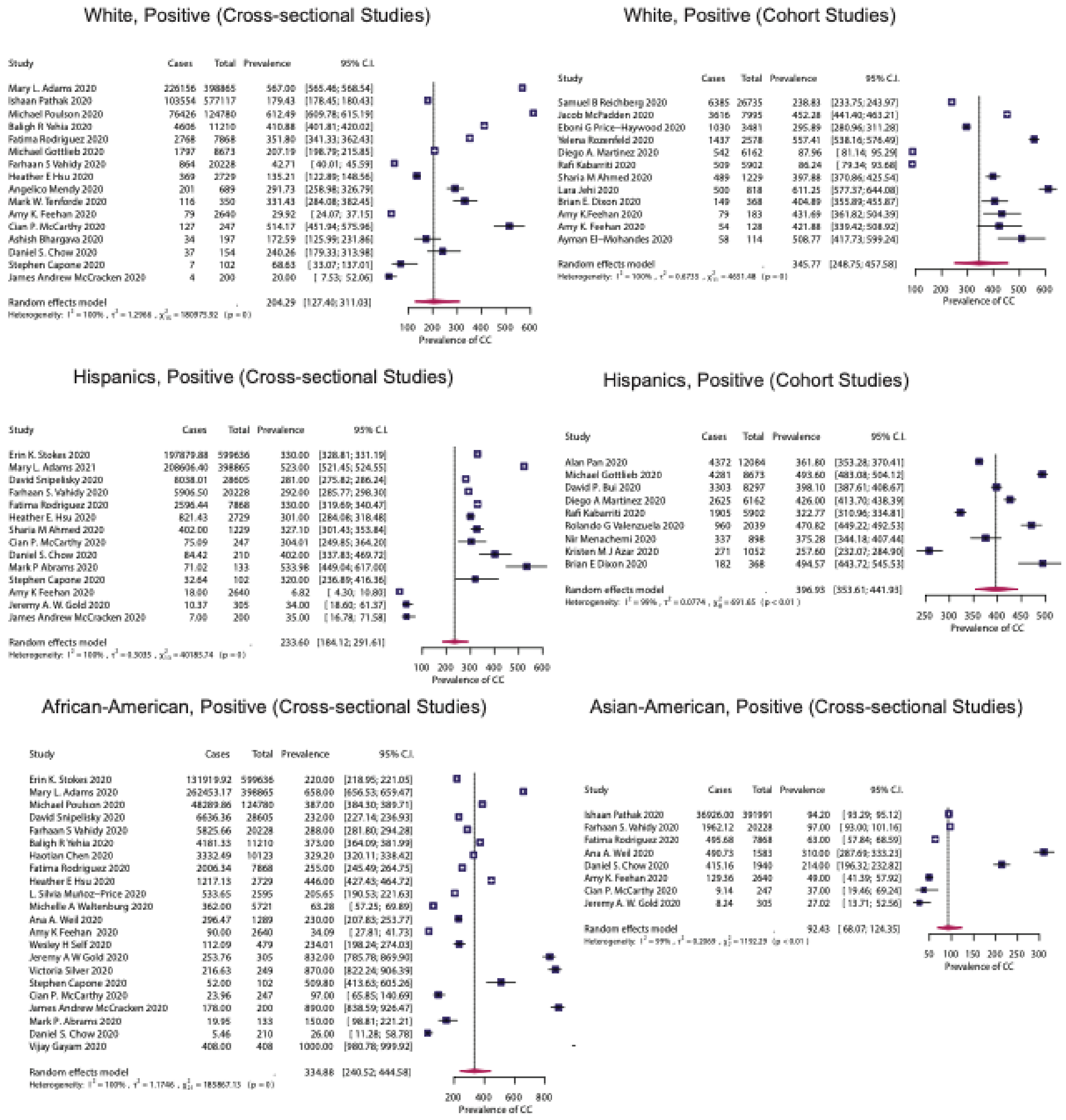

© 2021 Magesh S et al. *JAMA Network Open.*

eMethods 1. Methods Pertaining to Search Criteria and Data Collection

The following keywords were used to search by all fields, which includes full text, author name, journal name, and phrase, in each database: “COVID-19 AND race”, “COVID-19 AND ethnicity”, “COVID-19 AND Asian patients”, “COVID-19 AND Black patients”, “COVID-19 AND White patients”, “COVID-19 AND Hispanic/Latino patients”, “COVID-19 AND American Indian/Alaska Natives patients”, “COVID-19 AND Pacific Islander patients”, “COVID-19 AND multiracial patients”, “income AND COVID-19”; “socioeconomic status AND COVID-19”, and “employment AND COVID-19.”

We used both the keyword and Medical Subject Heading (MeSH) term for the following keywords to increase the scope of our systematic review and meta-analysis: “COVID-19 AND ethnicity (MeSH term: COVID-19 AND ethnic groups)”, “COVID-19 AND race (MeSH term: COVID-19 AND race factors)”, “socioeconomic status AND COVID-19 (MeSH term: COVID-19 AND social class)”. MeSH terms provide controlled vocabulary for searches in databases, such as Pubmed. We chose to use both the MeSH term and the non-MeSH term for these particular keywords, as the non-MeSH term yielded significantly more results than the MeSH term. MeSH terms could not be used for the following keywords, as they were not available on the database: “COVID-19 AND Asian patients”, “COVID-19 AND Black patients”, “COVID-19 AND White patients”, “COVID-19 AND Hispanic/Latino patients”, “COVID-19 AND American Indian/Alaska Natives patients”, “COVID-19 AND Pacific Islander patients”, and “COVID-19 AND multiracial patients”. MeSH terms were solely used for the following keywords: “income AND COVID-19” and “employment AND COVID-19”.

Our original keyword searches yielded 21,745 total results. Of these articles, 14,519 were unique (eFigure 1). We excluded studies based on Abstract if they met one of the following criteria: (1) The article is irrelevant for the study question or has insufficient data, (2) The article does not discuss an outcome that is of interest, (3) The article is published in a non-standard format and/or in a foreign language. Only studies with original clinical data were included. Following the Abstract review, we screened the full text of the remaining 287 articles. After subsequent full-text screening using the same 3 exclusion criteria, a total of 68 studies were included for data analysis.

Study and patient characteristics were collected, including the study type, location, mean and median age, total number of patients in the study, and medical comorbidities. Specifically, we extracted data for the following medical comorbidities and conditions which we observed to be commonly reported across various studies: smoking status (both former and current smokers), median body mass index (BMI), BMI over 40, cardiovascular disease (including other heart conditions such as coronary artery disease), hypertension, chronic obstructive pulmonary disease (COPD), diabetes mellitus or diabetes, and occurence of malignancy or cancer. For the purposes of this analysis, we considered Hispanics and Latinos as a single cohort. The studies included did

© 2021 Magesh S et al. *JAMA Network Open.*

not differentiate between various Asian populations, so many Asian populations were considered as a single cohort.

Following initial data review, we extracted the zip code, geographic location and/or congressional district from each study included in our meta-analysis in order to identify socioeconomic variables for subsequent analyses. In instances where congressional district information was not provided, we determined this information based on the zip code or geographic location of the study. From this extracted information, we obtained the following data for various measures of socioeconomic disparity from external websites for each study: (1) County median income and the percentage of each race in the district where the study was conducted was taken from the US Census Bureau's website at the congressional district level. (2) Area Deprivation Index (ADI) was evaluated with The University of Wisconsin's Neighborhood Atlas tool and was constructed based on geographic location. (3) Measures of social determinants of health, including the percent of the population under age 65 that are uninsured, ratio of population to primary care physicians, and rate of hospital stays for ambulatory-care sensitive conditions per 100,000 Medicare enrollees (preventable hospital stays), were evaluated with the County Health Rankings and Roadmaps tool at the congressional district level. Geographic variation and population density were assessed with the Urban Core Opportunity Index as reported in the Social Determinants of Health Atlas. Area unit of analysis was limited to specific location and address level for this particular tool. For county-wide studies, a broader measure of each of these social determinants was calculated, using averages of data from each of the locations indicated in the study.

© 2021 Magesh S et al. *JAMA Network Open.*

eMethods 2. Citations of Articles that Appeared to Meet Inclusion Criteria but Were Excluded

Excluded for non-standard format:

1. Bassett, M. T., Chen, J. T., & Krieger, N. (2020). Variation in racial/ethnic disparities in COVID-19 mortality by age in the United States: A cross-sectional study. PLoS medicine, 17(10), e1003402.
2. Hawkins, R. B., Charles, E. J., & Mehaffey, J. H. (2020). Socio-economic status and COVID-19–related cases and fatalities. Public health, 189, 129-134.
3. Egede, L. E., Walker, R. J., Garacci, E., & Raymond Sr, J. R. (2020). Racial/Ethnic Differences In COVID-19 Screening, Hospitalization, And Mortality In Southeast Wisconsin: Study examines racial/ethnic differences in COVID-19 screening, symptom presentation, hospitalization, and mortality among 31,549 adults tested for COVID-19 in Wisconsin. Health Affairs, 39(11), 1926-1934.
4. Abedi, V., Olulana, O., Avula, V., Chaudhary, D., Khan, A., Shahjouei, S., ... & Zand, R. (2020). Racial, economic, and health inequality and COVID-19 infection in the United States. Journal of racial and ethnic health disparities, 1-11.
5. Alnababteh, M., Drescher, G., Jayaram, L., Kohli, A., Hashmi, M., Hayat, F., ... & Zaaqoq, A. (2020). INVESTIGATING THE RELATIONSHIP BETWEEN RACE/ETHNICITY AND CLINICAL OUTCOMES IN COVID-19. Chest, 158(4), A2477.
6. Aleman, V. D., Fernandez, E. G., Varon, D., Surani, S., Gathe, J., & Varon, J. (2020). SOCIOECONOMIC DISPARITIES AS A DETERMINANT RISK FACTOR IN THE INCIDENCE OF COVID-19. Chest, 158(4), A1039.
7. El-Mohandes, A., Ratzan, S. C., Rauh, L., Ngo, V., Rabin, K., Kimball, S., ... & Freudenberg, N. (2020). COVID-19: A Barometer for Social Justice in New York City.
8. Xie, J., Zu, Y., Alkhatib, A., Pham, T. T., Gill, F., Jang, A., ... & Denson, J. L. (2021). Metabolic syndrome and COVID-19 mortality among adult black patients in New Orleans. Diabetes Care, 44(1), 188-193.
9. Clift, A. K., Coupland, C. A., Keogh, R. H., Diaz-Ordaz, K., Williamson, E., Harrison, E. M., ... & Hippisley-Cox, J. (2020). Living risk prediction algorithm (QCOVID) for risk of hospital admission and mortality from coronavirus 19 in adults: national derivation and validation cohort study. bmj, 371.
10. Sepulchre, E., Pittie, G., Stojkovic, V., Haesbroek, G., Crama, Y., Schyns, M., ... & Minon, J. M. (2020). Covid-19: contribution of clinical characteristics and laboratory features for early detection of patients with high risk of severe evolution. Acta Clinica Belgica, 1-7.
11. Baquero, B., Gonzalez, C., Ramirez, M., Chavez Santos, E., & Ornelas, I. J. (2020). Understanding and Addressing Latinx COVID-19 Disparities in Washington State. Health Education & Behavior, 47(6), 845-849.
12. Le, T. K., Cha, L., Han, H. R., & Tseng, W. (2020). Anti-Asian Xenophobia and Asian American COVID-19 Disparities.

© 2021 Magesh S et al. *JAMA Network Open.*

13. Kim, H. N., Lan, K. F., Nkyekyer, E., Neme, S., Pierre-Louis, M., Chew, L., & Duber, H. C. (2020). Assessment of disparities in COVID-19 testing and infection across language groups in Seattle, Washington. JAMA network open, 3(9), e2021213-e2021213.
14. Hamidi, S., Ewing, R., & Sabouri, S. (2020). Longitudinal analyses of the relationship between development density and the COVID-19 morbidity and mortality rates: Early evidence from 1,165 metropolitan counties in the United States. Health & place, 64, 102378.
15. Rader, B., Astley, C. M., Sy, K. T. L., Sewalk, K., Hswen, Y., Brownstein, J. S., & Kraemer, M. U. (2020). Geographic access to United States SARS-CoV-2 testing sites highlights healthcare disparities and may bias transmission estimates.
16. He, J., He, L., Zhou, W., Nie, X., & He, M. (2020). Discrimination and social exclusion in the outbreak of COVID-19. International Journal of Environmental Research and Public Health, 17(8), 2933.
17. Do, D. P., & Frank, R. (2021). Unequal burdens: assessing the determinants of elevated COVID-19 case and death rates in New York City's racial/ethnic minority neighbourhoods. J Epidemiol Community Health, 75(4), 321-326.
18. Dasgupta, S., Bowen, V. B., Leidner, A., Fletcher, K., Musial, T., Rose, C., ... & Oster, A. M. (2020). Association Between Social Vulnerability and a County's Risk for Becoming a COVID-19 Hotspot—United States, June 1–July 25, 2020. Morbidity and Mortality Weekly Report, 69(42), 1535.
19. Barasa, S. (2020). The major predictors of testing positive for COVID-19 among symptomatic hospitalized patients. medRxiv.
20. Benussi, A., Pilotto, A., Premi, E., Libri, I., Giunta, M., Agosti, C., ... & Padovani, A. (2020). Clinical characteristics and outcomes of inpatients with neurologic disease and COVID-19 in Brescia, Lombardy, Italy. Neurology, 95(7), e910-e920.
21. Bertsimas, D., Lukin, G., Mingardi, L., Nohadani, O., Orfanoudaki, A., Stellato, B., ... & Hellenic COVID-19 Study Group. (2020). COVID-19 mortality risk assessment: An international multi-center study. PloS one, 15(12), e0243262.
22. Bhowmik, T., Tirtha, S. D., Iraganaboina, N. C., & Eluru, N. (2021). A comprehensive analysis of COVID-19 transmission and mortality rates at the county level in the United States considering socio-demographics, health indicators, mobility trends and health care infrastructure attributes. Plos one, 16(4), e0249133.
23. Bilal, U., Barber, S., Tabb, L., & Diez-Roux, A. V. (2020). Spatial Inequities in COVID-19 Testing, Positivity, Incidence and Mortality in 3 US Cities: a Longitudinal Ecological Study. medRxiv.
24. Burden, S. J., Rademaker, J., Weedon, B. D., Whaymand, L., Dawes, H., & Jones, A. (2020). Associations of Global Country Profiles and Modifiable Risk Factors with COVID-19 Cases and Deaths. Available at SSRN 3627258.

© 2021 Magesh S et al. *JAMA Network Open.*

25. Cao, Y., Hiyoshi, A., & Montgomery, S. (2020). COVID-19 case-fatality rate and demographic and socioeconomic influencers: worldwide spatial regression analysis based on country-level data. BMJ open, 10(11), e043560.
26. Incerti, D., Rizzo, S., Li, X., Lindsay, L., Yau, V., Keebler, D., ... & Tsai, L. (2020). Risk factors for mortality among hospitalized patients with COVID-19. medRxiv.
27. Kiaghadi, A., Rifai, H. S., & Liaw, W. (2020). Assessing COVID-19 risk, vulnerability and infection prevalence in communities. Plos one, 15(10), e0241166.
28. Kranjac, A. W., & Kranjac, D. (2020). County-level factors influence the trajectory of Covid-19 incidence. medRxiv.
29. Robertson, L. S. (2020). COVID-19 Confirmed Cases and Fatalities in 883 US Counties with a Population of 50,000 or More: Predictions Based on Social, Economic, Demographic Factors and Shutdown Days. medRxiv.
30. Lieberman-Cribbin, W., Tuminello, S., Flores, R. M., & Taioli, E. (2020). Disparities in COVID-19 testing and positivity in New York City. American journal of preventive medicine, 59(3), 326-332.
31. Center, K. E., Da Silva, J., Hernandez, A. L., Vang, K., Martin, D. W., Mazurek, J., ... & James, A. E. (2020). Multidisciplinary community-based investigation of a COVID-19 outbreak among Marshallese and Hispanic/Latino communities—Benton and Washington Counties, Arkansas, March–June 2020. Morbidity and Mortality Weekly Report, 69(48), 1807.

Excluded because desired outcomes were not measured:

1. Gross, C. P., Essien, U. R., Pasha, S., Gross, J. R., Wang, S. Y., & Nunez-Smith, M. (2020). Racial and ethnic disparities in population-level Covid-19 mortality. Journal of general internal medicine, 35(10), 3097-3099.
2. Richmond, H. L., Tome, J., Rochani, H., Fung, I. C. H., Shah, G. H., & Schwind, J. S. (2020). The Use of Penalized Regression Analysis to Identify County-Level Demographic and Socioeconomic Variables Predictive of Increased COVID-19 Cumulative Case Rates in the State of Georgia. International journal of environmental research and public health, 17(21), 8036.
3. Oronce, C. I. A., Scannell, C. A., Kawachi, I., & Tsugawa, Y. (2020). Association between state-level income inequality and COVID-19 cases and mortality in the USA. Journal of General Internal Medicine, 35(9), 2791-2793.
4. El Chaar, M., King, K., & Lima, A. G. (2020). Are black and Hispanic persons disproportionately affected by COVID-19 because of higher obesity rates?. Surgery for Obesity and Related Diseases, 16(8), 1096-1099.
5. Maroko, A. R., Nash, D., & Pavilonis, B. T. (2020). Covid-19 and Inequity: A comparative spatial analysis of New York City and Chicago hot spots. Journal of Urban Health, 97(4), 461-470.

© 2021 Magesh S et al. *JAMA Network Open.*

6. Kaushik, S., Aydin, S. I., Derespina, K. R., Bansal, P. B., Kowalsky, S., Trachtman, R., ... & Medar, S. S. (2020). Multisystem inflammatory syndrome in children (MIS-C) associated with SARS-CoV-2 infection: a multi-institutional study from New York City. The Journal of Pediatrics.

Excluded due to insufficient data:

1. Holmes, L., Enwere, M., Williams, J., Ogundele, B., Chavan, P., Piccoli, T., ... & Dabney, K. W. (2020). Black–White risk differentials in COVID-19 (SARS-COV2) transmission, mortality and case fatality in the United States: translational epidemiologic perspective and challenges. International journal of environmental research and public health, 17(12), 4322.
2. Rodriguez-Lonebear, D., Barceló, N. E., Akee, R., & Carroll, S. R. (2020). Research Full Report: American Indian Reservations and COVID-19: Correlates of Early Infection Rates in the Pandemic. Journal of Public Health Management and Practice, 26(4), 371.
3. Rogers, T. N., Rogers, C. R., VanSant?Webb, E., Gu, L. Y., Yan, B., & Qeadan, F. (2020). Racial Disparities in COVID?19 Mortality Among Essential Workers in the United States. World medical & health policy, 12(3), 311-327.
4. Figueroa, J. F., Wadhera, R. K., Lee, D., Yeh, R. W., & Sommers, B. D. (2020). Community-Level Factors Associated With Racial And Ethnic Disparities In COVID-19 Rates In Massachusetts: Study examines community-level factors associated with racial and ethnic disparities in COVID-19 rates in Massachusetts. Health affairs, 39(11), 1984-1992.
5. Krieger, N., Waterman, P. D., & Chen, J. T. (2020). COVID-19 and overall mortality inequities in the surge in death rates by ZIP Code characteristics: Massachusetts, January 1 to May 19, 2020. American Journal of Public Health, 110(12), 1850-1852.
6. Hawkins, D. (2020). Social determinants of COVID-19 in Massachusetts, United States: an ecological study. Journal of Preventive Medicine and Public Health, 53(4), 220.
7. Okoh, A. K., Sossou, C., Dangayach, N. S., Meledathu, S., Phillips, O., Raczek, C., ... & Grewal, H. S. (2020). Coronavirus disease 19 in minority populations of Newark, New Jersey. International journal for equity in health, 19(1), 1-8.
8. Ramírez, I. J., & Lee, J. (2020). COVID-19 emergence and social and health determinants in Colorado: a rapid spatial analysis. International journal of environmental research and public health, 17(11), 3856.
9. Raine, S., Liu, A., Mintz, J., Wahood, W., Huntley, K., & Haffizulla, F. (2020). Racial and ethnic disparities in COVID-19 outcomes: social determination of health. International journal of environmental research and public health, 17(21), 8115.
10. Gross, C. P., Essien, U. R., Pasha, S., Gross, J. R., Wang, S. Y., & Nunez-Smith, M. (2020). Racial and ethnic disparities in population-level Covid-19 mortality. Journal of general internal medicine, 35(10), 3097-3099.

© 2021 Magesh S et al. *JAMA Network Open.*

11. Khan, A., Chatterjee, A., & Singh, S. (2020). Comorbidities and disparities in outcomes of COVID-19 among African American and White patients. medRxiv.
12. Bryan, M. S., Sun, J., Jagai, J., Horton, D. E., Montgomery, A., Sargis, R., & Argos, M. (2021). Coronavirus disease 2019 (COVID-19) mortality and neighborhood characteristics in Chicago. Annals of epidemiology, 56, 47-54.
13. Cowger, T. L., Davis, B. A., Etkins, O. S., Makofane, K., Lawrence, J. A., Bassett, M. T., & Krieger, N. (2020). Comparison of weighted and unweighted population data to assess inequities in coronavirus disease 2019 deaths by race/ethnicity reported by the US Centers for Disease Control and Prevention. JAMA network open, 3(7), e2016933-e2016933.
14. Ruprecht, M. M., Wang, X., Johnson, A. K., Xu, J., Felt, D., Ihenacho, S., ... & Phillips Ii, G. (2021). Evidence of social and structural COVID-19 disparities by sexual orientation, gender identity, and race/ethnicity in an urban environment. Journal of Urban Health, 98(1), 27-40.
15. Mahajan, U. V., & Larkins-Pettigrew, M. (2020). Racial demographics and COVID-19 confirmed cases and deaths: a correlational analysis of 2886 US counties. Journal of Public Health, 42(3), 445-447.
16. Feldman, J. M., & Bassett, M. T. (2020). The relationship between neighborhood poverty and COVID-19 mortality within racial/ethnic groups (Cook County, Illinois). medRxiv.
17. Mukherji, N. (2020). The social and economic factors underlying the incidence of COVID-19 cases and deaths in US counties. MedRxiv.
18. Paul, A., Englert, P., & Varga, M. (2020). Socio-economic disparities and COVID-19 in the USA. arXiv preprint arXiv:2009.04935.
19. Bertocchi, G., & Dimico, A. (2020). COVID-19, race, and redlining.
20. Cyrus, E., Clarke, R., Hadley, D., Bursac, Z., Trepka, M. J., Dévieux, J. G., ... & Wagner, E. F. (2020). The impact of COVID-19 on African American communities in the United States. Health equity, 4(1), 476-483.
21. Gupta, R., Agrawal, R., Bukhari, Z., Jabbar, A., Wang, D., Diks, J., ... & Haseeb, M. A. (2020). Higher Comorbidities and Early Death is Characteristic of Hospitalized African-American Patients with COVID-19. medRxiv.
22. Lobelo, F., Bienvenida, A. X., Leung, S., Mbanya, A. N., Leslie, E. J., Koplan, K. E., & Shin, S. R. (2020). Clinical, Behavioral and Social Factors Associated with Racial Disparities in Hospitalized and Ambulatory COVID-19 Patients from an Integrated Health Care System in Georgia. medRxiv.
23. Hawkins, R. B., Charles, E. J., & Mehaffey, J. H. (2020). Socio-economic status and COVID-19–related cases and fatalities. Public health, 189, 129-134.
24. Ahmed, R., Williamson, M., Hamid, M. A., & Ashraf, N. (2020, September). United States County-level COVID-19 Death Rates and Case Fatality Rates Vary by Region and Urban Status. In Healthcare (Vol. 8, No. 3, p. 330). Multidisciplinary Digital Publishing Institute.

© 2021 Magesh S et al. *JAMA Network Open.*

25. Millett, G. A., Jones, A. T., Benkeser, D., Baral, S., Mercer, L., Beyrer, C., ... & Sullivan, P. S. (2020). Assessing differential impacts of COVID-19 on black communities. Annals of epidemiology, 47, 37-44.
26. Ahmad, K., Erqou, S., Shah, N., Nazir, U., Morrison, A. R., Choudhary, G., & Wu, W. C. (2020). Association of poor housing conditions with COVID-19 incidence and mortality across US counties. PloS one, 15(11), e0241327.
27. DiMaggio, C., Klein, M., Berry, C., & Frangos, S. (2020). Black/African American Communities are at highest risk of COVID-19: spatial modeling of New York City ZIP Code–level testing results. Annals of epidemiology, 51, 7-13.
28. Izzy, S., Tahir, Z., Cote, D. J., Al Jarrah, A., Roberts, M. B., Turbett, S., ... & El Khoury, J. (2020, October). Characteristics and Outcomes of Latinx Patients With COVID-19 in Comparison With Other Ethnic and Racial Groups. In Open forum infectious diseases (Vol. 7, No. 10, p. ofaa401). US: Oxford University Press.
29. Cheng, K. J. G., Sun, Y., & Monnat, S. M. (2020). COVID?19 Death Rates Are Higher in Rural Counties With Larger Shares of Blacks and Hispanics. The Journal of Rural Health, 36(4), 602-608.
30. Tirupathi, R., Muradova, V., Shekhar, R., Salim, S. A., Al-Tawfiq, J. A., & Palabindala, V. (2020). COVID-19 disparity among racial and ethnic minorities in the US: A cross sectional analysis. Travel medicine and infectious disease, 38, 101904.
31. Lamb, M. R., Kandula, S., & Shaman, J. (2021). Differential COVID?19 case positivity in New York City neighborhoods: Socioeconomic factors and mobility. Influenza and Other Respiratory Viruses, 15(2), 209-217.
32. Moore, J. T., Ricaldi, J. N., Rose, C. E., Fuld, J., Parise, M., Kang, G. J., ... & Honein, M. A. (2020). Disparities in incidence of COVID-19 among underrepresented racial/ethnic groups in counties identified as hotspots during June 5–18, 2020—22 states, February–June 2020. Morbidity and Mortality Weekly Report, 69(33), 1122.
33. Rentsch, C. T., Kidwai-Khan, F., Tate, J. P., Park, L. S., King Jr, J. T., Skanderson, M., ... & Justice, A. C. (2020). Patterns of COVID-19 testing and mortality by race and ethnicity among United States veterans: A nationwide cohort study. PLoS medicine, 17(9), e1003379.
34. Chen, J. T., & Krieger, N. (2020). Revealing the unequal burden of COVID-19 by income, race/ethnicity, and household crowding: US county versus zip code analyses. Journal of Public Health Management and Practice, 27(1), S43-S56.
35. Golestaneh, L., Neugarten, J., Fisher, M., Billett, H. H., Gil, M. R., Johns, T., ... & Bellin, E. (2020). The association of race and COVID-19 mortality. EClinicalMedicine, 25, 100455.
36. Chang, H. Y., Tang, W., Hatef, E., Kitchen, C., Weiner, J. P., & Kharrazi, H. (2020). Differential Impact of Mitigation Policies and Socioeconomic Status on COVID-19 Prevalence and Social Distancing in the United States. medRxiv.

© 2021 Magesh S et al. *JAMA Network Open.*

37. Wortham, J. M. (2020). Characteristics of persons who died with COVID-19—United States, February 12–May 18, 2020. MMWR. Morbidity and mortality weekly report, 69.
38. Parpia, A. S., Martinez, I., El-Sayed, A. M., Wells, C. R., Myers, L., Duncan, J., ... & Pandey, A. (2021). Racial disparities in COVID-19 mortality across Michigan, United States. EClinicalMedicine, 33, 100761.
39. Whittle, R. S., & Diaz-Artiles, A. (2020). An ecological study of socioeconomic predictors in detection of COVID-19 cases across neighborhoods in New York City. BMC medicine, 18(1), 1-17.
40. DiMaggio, C., Klein, M., Berry, C., & Frangos, S. (2020). Black/African American Communities are at highest risk of COVID-19: spatial modeling of New York City ZIP Code–level testing results. Annals of epidemiology, 51, 7-13.
41. Ahmed, S. M., Shah, R. U., Bale, M., Peacock, J. B., Berger, B., Brown, A., ... & Keegan, L. T. (2020). Comprehensive testing highlights racial, ethnic, and age disparities in the COVID-19 outbreak. medRxiv.
42. King Jr, J. T., Yoon, J. S., Rentsch, C. T., Tate, J. P., Park, L. S., Kidwai-Khan, F., ... & Justice, A. C. (2020). Development and validation of a 30-day mortality index based on pre-existing medical administrative data from 13,323 COVID-19 patients: The Veterans Health Administration COVID-19 (VACO) Index. PloS one, 15(11), e0241825.
43. Pasco, R. F., Fox, S. J., Johnston, S. C., Pignone, M., & Meyers, L. A. (2020). Estimated association of construction work with risks of COVID-19 infection and hospitalization in Texas. JAMA network open, 3(10), e2026373-e2026373.
44. Kaufman, H. W., Niles, J. K., & Nash, D. B. (2021). Disparities in SARS-CoV-2 positivity rates: associations with race and ethnicity. Population health management, 24(1), 20-26.
45. Razavi, A. C., Kelly, T. N., He, J., Fernandez, C., Whelton, P. K., Krousel?Wood, M., & Bazzano, L. A. (2020). Cardiovascular disease prevention and implications of coronavirus disease 2019: an evolving case study in the Crescent city. Journal of the American Heart Association, 9(13), e016997.
46. Ioannou, G. N., Locke, E., Green, P., Berry, K., O'Hare, A. M., Shah, J. A., ... & Fan, V. S. (2020). Risk factors for hospitalization, mechanical ventilation, or death among 10 131 US veterans with SARS-CoV-2 infection. JAMA network open, 3(9), e2022310-e2022310.
47. Bailey, L. C., Razzaghi, H., Burrows, E. K., Bunnell, H. T., Camacho, P. E., Christakis, D. A., ... & Forrest, C. B. (2021). Assessment of 135 794 pediatric patients tested for severe acute respiratory syndrome coronavirus 2 across the United States. JAMA pediatrics, 175(2), 176-184.
48. Reichberg, S. B., Mitra, P. P., Haghamad, A., Ramrattan, G., Crawford, J. M., Northwell COVID-19 Research Consortium, ... & Ziemba, Y. C. (2020). Rapid emergence of SARS-CoV-2 in the greater New York metropolitan area: geolocation, demographics,

© 2021 Magesh S et al. *JAMA Network Open.*

positivity rates, and hospitalization for 46 793 persons tested by northwell health. Clinical Infectious Diseases, 71(12), 3204-3213.

49. Thompson, C. N., Baumgartner, J., Pichardo, C., Toro, B., Li, L., Arciuolo, R., ... & Fine, A. (2020). COVID-19 Outbreak—New York City, February 29–June 1, 2020. Morbidity and Mortality Weekly Report, 69(46), 1725.
50. Killerby, M. E., Link-Gelles, R., Haight, S. C., Schrodt, C. A., England, L., Gomes, D. J., ... & Wong, K. K. (2020). Characteristics associated with hospitalization among patients with COVID-19—Metropolitan Atlanta, Georgia, March–April 2020. Morbidity and Mortality Weekly Report, 69(25), 790.
51. Labgold, K., Hamid, S., Shah, S., Gandhi, N. R., Chamberlain, A., Khan, F., ... & Collin, L. J. (2020). Widening the gap: greater racial and ethnic disparities in COVID-19 burden after accounting for missing race/ethnicity data. Medrxiv.
52. Cates, J., Lucero-Obusan, C., Dahl, R. M., Schirmer, P., Garg, S., Oda, G., ... & Cardemil, C. V. (2020). Risk for In-Hospital Complications Associated with COVID-19 and Influenza—Veterans Health Administration, United States, October 1, 2018–May 31, 2020. Morbidity and Mortality Weekly Report, 69(42), 1528.
53. Kim, L., Garg, S., O'Halloran, A., Whitaker, M., Pham, H., Anderson, E. J., ... & Langley, G. E. (2020). Risk factors for intensive care unit admission and in-hospital mortality among hospitalized adults identified through the US coronavirus disease 2019 (COVID-19)-associated hospitalization surveillance network (COVID-NET). Clinical Infectious Diseases.
54. Kalayjian, B. C., Conner, K., Butler, I., Myers, L., Telleria, C., Panchang, D., & Halperin, J. (2020). Race, Heart Rate, and Temperature Are Strongly Associated With COVID-19 at a Community-based Clinic in New Orleans. Mayo Clinic Proceedings: Innovations, Quality & Outcomes, 4(6), 683-686.
55. van Holm, E. J., Wyczalkowski, C. K., & Dantzler, P. A. (2020). Neighborhood conditions and the initial outbreak of COVID-19: the case of Louisiana. Journal of Public Health (Oxford, England).
56. Thomas, M. D., Michaels, E. K., Darling-Hammond, S., Nguyen, T. T., Glymour, M. M., & Vittinghoff, E. (2020). Whites' County-Level Racial Bias, COVID-19 Rates, and Racial Inequities in the United States. International journal of environmental research and public health, 17(22), 8695.
57. Chin, T., Kahn, R., Li, R., Chen, J. T., Krieger, N., Buckee, C. O., ... & Kiang, M. V. (2020). US county-level characteristics to inform equitable COVID-19 response. MedRxiv.
58. Mikami, T., Miyashita, H., Yamada, T., Harrington, M., Steinberg, D., Dunn, A., & Siau, E. (2021). Risk factors for mortality in patients with COVID-19 in New York City. Journal of general internal medicine, 36(1), 17-26.
59. Douglas, J. A., & Subica, A. M. (2020). COVID-19 treatment resource disparities and social disadvantage in New York City. Preventive medicine, 141, 106282.

© 2021 Magesh S et al. *JAMA Network Open.*

60. Wiemers, E. E., Abrahams, S., AlFakhri, M., Hotz, V. J., Schoeni, R. F., & Seltzer, J. A. (2020). Disparities in vulnerability to severe complications from COVID-19 in the United States (No. w27294). National Bureau of Economic Research.
61. Russell, F. M., Wang, A., Ehrman, R. R., Jacobs, J., Croft, A., & Larsen, C. (2020). Risk factors associated with hospital admission in COVID-19 patients initially admitted to an observation unit. The American Journal of Emergency Medicine.
62. Correa-Agudelo, E., Mersha, T., Hernandez, A., Branscum, A. J., MacKinnon, N. J., & Cuadros, D. F. (2020). Identification of Vulnerable Populations and Areas at Higher Risk of COVID-19 Related Mortality in the US. medRxiv.
63. Cunningham, G. B., & Wigfall, L. T. (2020). Race, explicit racial attitudes, implicit racial attitudes, and COVID-19 cases and deaths: An analysis of counties in the United States. PloS one, 15(11), e0242044.
64. Ko, J. Y., Danielson, M. L., Town, M., Derado, G., Greenlund, K. J., Kirley, P. D., ... & COVID-NET Surveillance Team. (2020). Risk Factors for Coronavirus Disease 2019 (COVID-19)–Associated Hospitalization: COVID-19–Associated Hospitalization Surveillance Network and Behavioral Risk Factor Surveillance System. Clinical Infectious Diseases.
65. Abedi, V., Olulana, O., Avula, V., Chaudhary, D., Khan, A., Shahjouei, S., ... & Zand, R. (2020). Racial, economic, and health inequality and COVID-19 infection in the United States. Journal of racial and ethnic health disparities, 1-11.
66. Ojinnaka, C. O., Adepoju, O. E., Burgess, A. V., & Woodard, L. (2020). Factors associated with COVID-Related mortality: the case of Texas. Journal of racial and ethnic health disparities, 1-6.
67. Ebinger, J. E., Achamallah, N., Ji, H., Claggett, B. L., Sun, N., Botting, P., ... & Cheng, S. (2020). Pre-existing traits associated with Covid-19 illness severity. PloS one, 15(7), e0236240.
68. Mani, V. R., Kalabin, A., Valdivieso, S. C., Murray-Ramcharan, M., & Donaldson, B. (2020). New York Inner City Hospital COVID-19 Experience and Current Data: Retrospective Analysis at the Epicenter of the American Coronavirus Outbreak. Journal of medical Internet research, 22(9), e20548.
69. Fox, T., Ruddiman, K., Lo, K. B., Peterson, E., DeJoy, R., Salacup, G., ... & Patarroyo-Aponte, G. (2021). The relationship between diabetes and clinical outcomes in COVID-19: a single-center retrospective analysis. Acta Diabetologica, 58(1), 33-38.
70. Ahmad, I., Jeyarajah, J., Nair, G., Ragbourne, S. C., Vowles, B., Wong, D. J., & El-Boghdadly, K. (2021). A prospective, observational, cohort study of airway management of patients with COVID-19 by specialist tracheal intubation teams. Canadian Journal of Anesthesia/Journal canadien d'anesthésie, 68(2), 196-203.
71. Hughes, M. M., Groenewold, M. R., Lessem, S. E., Xu, K., Ussery, E. N., Wiegand, R. E., ... & Stuckey, M. J. (2020). Update: characteristics of health care personnel with

COVID-19—United States, February 12–July 16, 2020. Morbidity and Mortality Weekly Report, 69(38), 1364.

72. Joseph, N. P., Reid, N. J., Som, A., Li, M. D., Hyle, E. P., Dugdale, C. M., ... & Flores, E. J. (2020). Racial and Ethnic Disparities in Disease Severity on Admission Chest Radiographs among Patients Admitted with Confirmed Coronavirus Disease 2019: A Retrospective Cohort Study. Radiology, 297(3), E303-E312.
73. Kim, L., Whitaker, M., O'Halloran, A., Kambhampati, A., Chai, S. J., Reingold, A., ... & COVID-NET Surveillance Team. (2020). Hospitalization rates and characteristics of children aged< 18 years hospitalized with laboratory-confirmed COVID-19—COVID-NET, 14 States, March 1–July 25, 2020. Morbidity and Mortality Weekly Report, 69(32), 1081.
74. James, M. K., Kishore, M., & Lee, S. W. (2020). Demographic and Socioeconomic Characteristics of COVID-19 Patients Treated in the Emergency Department of a New York City Hospital. Journal of community health, 1-8.
75. Tang, O., Bigelow, B. F., Sheikh, F., Peters, M., Zenilman, J. M., Bennett, R., & Katz, M. J. (2020). Outcomes of Nursing Home COVID-19 Patients by Initial Symptoms and Comorbidity: Results of Universal Testing of 1970 Residents. Journal of the American Medical Directors Association, 21(12), 1767-1773.
76. Miller, J., Fadel, R. A., Tang, A., Perrotta, G., Herc, E., Soman, S., ... & Suleyman, G. (2020). The Impact of Sociodemographic Factors, Comorbidities, and Physiologic Responses on 30-Day Mortality in Coronavirus Disease 2019 (COVID-19) Patients in Metropolitan Detroit. Clinical Infectious Diseases.
77. Bhayani, S., Sengupta, R., Markossian, T., Tootooni, S., Luke, A., Shoham, D., ... & Kramer, H. (2020). Dialysis, COVID-19, poverty, and race in greater Chicago: an ecological analysis. Kidney medicine, 2(5), 552-558.
78. Salerno, S., Zhao, Z., Sankar, S. P., Salvatore, M., Gu, T., Fritsche, L. G., ... & Mukherjee, B. (2020). Understanding the patterns of repeated testing for COVID-19: Association with patient characteristics and outcomes. medRxiv.
79. Campos-Castillo, C., & Anthony, D. (2021). Racial and ethnic differences in self-reported telehealth use during the COVID-19 pandemic: a secondary analysis of a US survey of internet users from late March. Journal of the American Medical Informatics Association, 28(1), 119-125.
80. Derespina, K. R., Kaushik, S., Plichta, A., Conway Jr, E. E., Bercow, A., Choi, J., ... & Medar, S. S. (2020). Clinical manifestations and outcomes of critically ill children and adolescents with coronavirus disease 2019 in New York city. The Journal of pediatrics, 226, 55-63.
81. Hernandez-Romieu, A. C., Adelman, M. W., Hockstein, M. A., Robichaux, C. J., Edwards, J. A., Fazio, J. C., ... & Auld, S. C. (2020). Timing of intubation and mortality among critically ill coronavirus disease 2019 patients: a single-center cohort study. Critical care medicine.

© 2021 Magesh S et al. *JAMA Network Open.*

82. Baker, J. M., Nelson, K. N., Overton, E., Lopman, B. A., Lash, T. L., Photakis, M., ... & Steinberg, J. P. (2020). Quantification of occupational and community risk factors for SARS-CoV-2 seropositivity among healthcare workers in a large US healthcare system. medRxiv.
83. Jean-Louis, G., Turner, A. D., Jin, P., Liu, M., Boutin-Foster, C., McFarlane, S. I., & Seixas, A. (2020). Increased metabolic burden among blacks: a putative mechanism for disparate Covid-19 Outcomes. Diabetes, metabolic syndrome and obesity: targets and therapy, 13, 3471.
84. Raad, M., Dabbagh, M., Gorgis, S., Yan, J., Chehab, O., Dagher, C., ... & Parikh, S. (2020). Cardiac injury patterns and inpatient outcomes among patients admitted with COVID-19. The American journal of cardiology, 133, 154-161.
85. Carethers, J. M. (2021). Insights into disparities observed with COVID?19. Journal of internal medicine, 289(4), 463-473.
86. Egede, L. E., & Walker, R. J. (2020). Structural racism, social risk factors, and Covid-19—A dangerous convergence for Black Americans. New England Journal of Medicine, 383(12), e77.
87. Ambrose, A. J. H. (2020). Inequities during COVID-19. Pediatrics, 146(2).
88. Quandt, S. A., LaMonto, N. J., Mora, D. C., Talton, J. W., Laurienti, P. J., & Arcury, T. A. (2020). COVID-19 Pandemic among Latinx Farmworker and Nonfarmworker Families in North Carolina: Knowledge, Risk Perceptions, and Preventive Behaviors. International journal of environmental research and public health, 17(16), 5786.
89. McGonagle, D., Plein, S., O'Donnell, J. S., Sharif, K., & Bridgewood, C. (2020). Increased cardiovascular mortality in African Americans with COVID-19. The Lancet Respiratory Medicine, 8(7), 649-651.
90. Tal, Y., Adini, A., Eran, A., & Adini, I. (2020). Racial disparity in Covid-19 mortality rates-A plausible explanation. Clinical Immunology (Orlando, Fla.).
91. Boserup, B., McKenney, M., & Elkbuli, A. (2020). Disproportionate Impact of COVID-19 Pandemic on Racial and Ethnic Minorities. The American Surgeon, 86(12), 1615-1622.
92. Gaffney, A. W., Hawks, L., Bor, D. H., Woolhandler, S., Himmelstein, D. U., & McCormick, D. (2020). 18.2 Million Individuals at Increased Risk of Severe COVID-19 Illness Are Un-or Underinsured. Journal of General Internal Medicine, 35(8), 2487-2489.
93. Chaturvedi, R., & Gabriel, R. A. (2020). Coronavirus disease health care delivery impact on African Americans. Disaster Medicine and Public Health Preparedness, 1-3.
94. Goldman, N., Pebley, A. R., Lee, K., Andrasfay, T., & Pratt, B. (2020). Racial and Ethnic Differentials in COVID-19-Related Job Exposures by Occupational Status in the US. medRxiv.
95. Feinhandler, I., Cilento, B., Beauvais, B., Harrop, J., & Fulton, L. (2020, September). Predictors of Death Rate during the COVID-19 Pandemic. In Healthcare (Vol. 8, No. 3, p. 339). Multidisciplinary Digital Publishing Institute.

© 2021 Magesh S et al. *JAMA Network Open.*

96. Sáenz, R., & Garcia, M. A. (2021). The disproportionate impact of COVID-19 on older Latino mortality: The rapidly diminishing Latino paradox. The Journals of Gerontology: Series B, 76(3), e81-e87.
97. Strully, K., Yang, T. C., & Liu, H. (2021). Regional variation in COVID-19 disparities: connections with immigrant and Latinx communities in US counties. Annals of epidemiology, 53, 56-62.
98. Kim, D., Adeniji, N., Latt, N., Kumar, S., Bloom, P. P., Aby, E. S., ... & Dhanasekaran, R. (2020). Predictors of outcomes of COVID-19 in patients with chronic liver disease: US multi-center study. Clinical Gastroenterology and Hepatology.
99. Ferrando-Vivas, P., Doidge, J., Thomas, K., Gould, D. W., Mouncey, P., Shankar-Hari, M., ... & Harrison, D. A. (2021). Prognostic factors for 30-day mortality in critically ill patients with coronavirus disease 2019: an observational cohort study. Critical care medicine, 49(1), 102.
100. Bixler D, Miller AD, Mattison CP, et al. SARS-CoV-2–Associated Deaths Among Persons Aged <21 Years — United States, February 12–July 31, 2020. MMWR Morb Mortal Wkly Rep 2020;69:1324–1329. DOI: http://dx.doi.org/10.15585/mmwr.mm6937e4external icon
101. Yamaki, J., Peled, H., Mathews, S., Park, D., Firoozi, M., Smith, K., & Nguyen, L. (2020). Seroprevalence of Novel Coronavirus SARS-CoV-2 at a Community Hospital Emergency Department and Outpatient Laboratory in Northern Orange County, California. Journal of racial and ethnic health disparities, 1-5.
102. Adams, M. L., Katz, D. L., & Grandpre, J. (2020). Population based estimates of comorbidities affecting risk for complications from COVID-19 in the US. medRxiv.
103. Ahmad, K., Erqou, S., Shah, N., Nazir, U., Morrison, A. R., Choudhary, G., & Wu, W. C. (2020). Association of poor housing conditions with COVID-19 incidence and mortality across US counties. PloS one, 15(11), e0241327.
104. Chambless, L. (2020). Why do per capita COVID-19 Case Rates Differ Between US States?. medRxiv.
105. Chow, D. S., Soun, J., Gavis-Bloom, J., Weinberg, B., Chang, P., Mutasa, S., ... & Boden-Albala, B. (2020). The disproportionate rise in COVID-19 cases among Hispanic/Latinx in disadvantaged communities of Orange County, California: A socioeconomic case-series. medrxiv.
106. Davis, C., Gao, M., Nichols, M., & Henao, R. (2020). Predicting Hospital Utilization and Inpatient Mortality of Patients Tested for COVID-19. medRxiv.
107. Kathe, N., & Wani, R. J. (2020). Determinants of COVID-19 Incidence and Mortality in the US: Spatial Analysis. medRxiv.
108. Khan, S., McCabe, M., Krefman, A., Petito, L. C., Yang, X., Kershaw, K., ... & Allen, N. B. (2020). A county-level susceptibility index and coronavirus disease 2019 mortality in the united states: A socioecological study. medRxiv.

© 2021 Magesh S et al. *JAMA Network Open.*

109. Feehan, A. K., Fort, D., Garcia-Diaz, J., Price-Haywood, E. G., Velasco, C., Sapp, E., ... & Seoane, L. (2020). Seroprevalence of SARS-CoV-2 and infection fatality ratio, Orleans and Jefferson parishes, Louisiana, USA, May 2020. Emerging infectious diseases, 26(11), 2765.
110. Seehuus, M., Stanton, A. M., Handy, A. B., Haik, A. K., Gorman, R., & Clifton, J. (2021). Impact of COVID-19 predicts perceived risk more strongly than known demographic risk factors. Journal of Psychosomatic Research, 140, 110299.
111. Bhargava, A., Fukushima, E. A., Levine, M., Zhao, W., Tanveer, F., Szpunar, S. M., & Saravolatz, L. (2020). Predictors for severe COVID-19 infection. Clinical Infectious Diseases, 71(8), 1962-1968.
112. Jehi, L., Ji, X., Milinovich, A., Erzurum, S., Merlino, A., Gordon, S., ... & Kattan, M. W. (2020). Development and validation of a model for individualized prediction of hospitalization risk in 4,536 patients with COVID-19. PloS one, 15(8), e0237419.
113. Thomas, M. D., Michaels, E. K., Darling-Hammond, S., Nguyen, T. T., Glymour, M. M., & Vittinghoff, E. (2020). Whites' County-Level Racial Bias, COVID-19 Rates, and Racial Inequities in the United States. International journal of environmental research and public health, 17(22), 8695.
114. Salvatore, P. P., Sula, E., Coyle, J. P., Caruso, E., Smith, A. R., Levine, R. S., ... & Bhattarai, A. (2020). Recent increase in COVID-19 cases reported among adults aged 18–22 years—United States, May 31–September 5, 2020. Morbidity and Mortality Weekly Report, 69(39), 1419.
115. Rosenberg, E. S., Tesoriero, J. M., Rosenthal, E. M., Chung, R., Barranco, M. A., Styer, L. M., ... & Zucker, H. A. (2020). Cumulative incidence and diagnosis of SARS-CoV-2 infection in New York. Annals of epidemiology, 48, 23-29.

Excluded due to low quality score:

1. Asare, S., Sandio, A., Opara, I. N., Riddle-Jones, L., Palla, M., Renny, N., & Ayers, E. (2020). Higher obesity trends among African Americans are associated with increased mortality in infected COVID-19 patients within the City of Detroit. SN Comprehensive Clinical Medicine, 2(8), 1045-1047.
2. Lieberman-Cribbin, W., Tuminello, S., Flores, R. M., & Taioli, E. (2020). Disparities in COVID-19 testing and positivity in New York City. American journal of preventive medicine, 59(3), 326-332.
3. Cheng, K. J. G., Sun, Y., & Monnat, S. M. (2020). COVID?19 Death Rates Are Higher in Rural Counties With Larger Shares of Blacks and Hispanics. The Journal of Rural Health, 36(4), 602-608.
4. Ogedegbe, G., Ravenell, J., Adhikari, S., Butler, M., Cook, T., Francois, F., ... & Horwitz, L. I. (2020). Assessment of racial/ethnic disparities in hospitalization and

© 2021 Magesh S et al. *JAMA Network Open.*

mortality in patients with COVID-19 in New York city. JAMA network open, 3(12), e2026881-e2026881.
5. Bui, D. P., McCaffrey, K., Friedrichs, M., LaCross, N., Lewis, N. M., Sage, K., ... & Dunn, A. (2020). Racial and ethnic disparities among COVID-19 cases in workplace outbreaks by industry sector—Utah, March 6–June 5, 2020. Morbidity and Mortality Weekly Report, 69(33), 1133.
6. Scannell, C. A., Oronce, C. I. A., & Tsugawa, Y. (2020). Association Between County-Level Racial and Ethnic Characteristics and COVID-19 Cases and Deaths in the USA. Journal of general internal medicine, 35(10), 3126-3128.
7. Adegunsoye, A., Ventura, I. B., & Liarski, V. M. (2020). Association of black race with outcomes in COVID-19 disease: a retrospective cohort study. Annals of the American Thoracic Society, 17(10), 1336-1339.
8. Martinez, D. A., Hinson, J. S., Klein, E. Y., Irvin, N. A., Saheed, M., Page, K. R., & Levin, S. R. (2020). SARS-CoV-2 positivity rate for Latinos in the Baltimore–Washington, DC Region. Jama, 324(4), 392-395.
9. Rentsch, C. T., Kidwai-Khan, F., Tate, J. P., Park, L. S., King Jr, J. T., Skanderson, M., ... & Justice, A. C. (2020). Covid-19 by race and ethnicity: a national cohort study of 6 million United States veterans. MedRxiv.
10. Sehra, S. T., Fundin, S., Lavery, C., & Baker, J. F. (2020). Differences in race and other state?level characteristics and associations with mortality from COVID?19 infection. Journal of medical virology, 92(11), 2406-2408.
11. Misa, N. Y., Perez, B., Basham, K., Fisher-Hobson, E., Butler, B., King, K., ... & Anderson, E. S. (2020). Racial/ethnic disparities in COVID-19 disease burden & mortality among emergency department patients in a safety net health system. The American journal of emergency medicine.
12. Takagi, H., Kuno, T., Yokoyama, Y., Ueyama, H., Matsushiro, T., Hari, Y., & Ando, T. (2021). Ethnicity/race and economics in COVID-19: meta-regression of data from counties in the New York metropolitan area. J Epidemiol Community Health, 75(2), 205-206.
13. Cromer, S. J., Lakhani, C. M., Wexler, D. J., Burnett-Bowie, S. A. M., Udler, M., & Patel, C. J. (2020). Geospatial Analysis of Individual and Community-Level Socioeconomic Factors Impacting SARS-CoV-2 Prevalence and Outcomes. Medrxiv.
14. Kitchen, C., Hatef, E., Chang, H. Y., Weiner, J., & Kharrazi, H. (2020). Assessing the Impact of Area Deprivation Index on COVID-19 Prevalence: A Contrast Between Rural and Urban US Jurisdictions. medRxiv.
15. Loomba, R. S., Aggarwal, G., Aggarwal, S., Flores, S., Villarreal, E. G., Farias, J. S., & Lavie, C. J. (2021). Disparities in case frequency and mortality of coronavirus disease 2019 (COVID-19) among various states in the United States. Annals of medicine, 53(1), 151-159.

16. Neelon, B., Mutiso, F., Mueller, N. T., Pearce, J. L., & Benjamin-Neelon, S. E. (2021). Spatial and temporal trends in social vulnerability and COVID-19 incidence and death rates in the United States. Plos one, 16(3), e0248702.
17. Riley, P., Riley, A., Turtle, J., & Ben-Nun, M. (2020). COVID-19 Deaths: Which Explanatory Variables Matter the Most?. medRxiv.
18. Tian, T., Zhang, J., Hu, L., Jiang, Y., Duan, C., Li, Z., ... & Zhang, H. (2021). Risk factors associated with mortality of COVID-19 in 3125 counties of the United States. Infectious diseases of poverty, 10(1), 1-8.
19. Debopadhaya, S., Sprague, A. D., Mou, H., Benavides, T. L., Ahn, S. M., Reschke, C. A., ... & Bennett, K. P. (2020). Social Determinants Associated with COVID-19 Mortality in the United States. medRxiv.
20. Neelon, B., Mutiso, F., Mueller, N. T., Pearce, J. L., & Benjamin-Neelon, S. E. (2020). Associations between governor political affiliation and COVID-19 cases and deaths in the United States. medRxiv.
21. Hu, T., Yue, H., Wang, C., She, B., Ye, X., Liu, R., ... & Bao, S. (2020). Racial Segregation, Testing Site Access, and COVID-19 Incidence Rate in Massachusetts, USA. International journal of environmental research and public health, 17(24), 9528.
22. Li, D., Gaynor, S. M., Quick, C., Chen, J. T., Stephenson, B. J., Coull, B. A., & Lin, X. (2020). Unraveling US National COVID-19 Racial/Ethnic Disparities using County Level Data Among 328 Million Americans. medRxiv.
23. Marcello, R. K., Dolle, J., Tariq, A., Kaur, S., Wong, L., Curcio, J., ... & Islam, N. (2020). Disaggregating Asian Race Reveals COVID-19 Disparities among Asian Americans at New York City's Public Hospital System. medRxiv.
24. Hawkins, D. (2020). Differential occupational risk for COVID?19 and other infection exposure according to race and ethnicity. American journal of industrial medicine, 63(9), 817-820.
25. Karaca-Mandic, P., Georgiou, A., & Sen, S. (2021). Assessment of COVID-19 hospitalizations by race/ethnicity in 12 states. JAMA internal medicine, 181(1), 131-134.
26. Fielding-Miller, R. K., Sundaram, M. E., & Brouwer, K. (2020). Social determinants of COVID-19 mortality at the county level. PloS one, 15(10), e0240151.
27. Guha, A., Bonsu, J., Dey, A., & Addison, D. (2020). Community and Socioeconomic Factors Associated with COVID-19 in the United States: Zip code level cross sectional analysis. medRxiv.
28. Takagi, H., Kuno, T., Yokoyama, Y., Ueyama, H., Matsushiro, T., Hari, Y., & Ando, T. (2020). Ethnics and economics in COVID-19: Meta-regression of data from countries in the New York metropolitan area. medRxiv.
29. Antwi-Amoabeng, D., Beutler, B. D., Awad, M., Kanji, Z., Mahboob, S., Ghuman, J., ... & Gullapalli, N. (2021). Sociodemographic predictors of outcomes in COVID-19: examining the impact of ethnic disparities in Northern Nevada. Cureus, 13(2).

© 2021 Magesh S et al. *JAMA Network Open.*

30. Retamales, V. A., Suarez, O. M., Lara-Garcia, O. E., Ranjha, S., Maini, R., Hingle, S., ... & Robinson, R. (2020). Racial/Ethnic Disparities in COVID-19 Hospital Admissions. medRxiv.
31. Betson, N., & Maitra, A. (2020). Disproportionate COVID-19 Related Mortality Amongst African Americans in Four Southern States in the United States. medRxiv.
32. Fabic, M. S., Choi, Y., & Bishai, D. (2020). Deaths among COVID Cases in the United States: Racial and Ethnic Disparities Persist. medRxiv.
33. Sy, K. T. L., Martinez, M. E., Rader, B., & White, L. F. (2020). Socioeconomic disparities in subway use and COVID-19 outcomes in New York City. MedRxiv.
34. Kim, S. J., & Bostwick, W. Social Vulnerability and Racial Inequality in COVID-19 Deaths in 508 Chicago. Health education & behavior: the official publication of the Society for Public Health, 509, 509-13.
35. Peters, D. J. (2020). Community susceptibility and resiliency to COVID?19 across the rural?urban continuum in the United States. The Journal of Rural Health, 36(3), 446-456.
36. Figueroa, J. F., Wadhera, R. K., Mehtsun, W. T., Riley, K., Phelan, J., & Jha, A. K. (2021, March). Association of race, ethnicity, and community-level factors with COVID-19 cases and deaths across US counties. In Healthcare (Vol. 9, No. 1, p. 100495). Elsevier.
37. Hatcher, S. M., Agnew-Brune, C., Anderson, M., Zambrano, L. D., Rose, C. E., Jim, M. A., ... & McCollum, J. (2020). COVID-19 Among American Indian and Alaska Native Persons—23 States, January 31–July 3, 2020. Morbidity and Mortality Weekly Report, 69(34), 1166.
38. Azar, K. M., Shen, Z., Romanelli, R. J., Lockhart, S. H., Smits, K., Robinson, S., ... & Pressman, A. R. (2020). Disparities In Outcomes Among COVID-19 Patients In A Large Health Care System In California: Study estimates the COVID-19 infection fatality rate at the US county level. Health Affairs, 39(7), 1253-1262.
39. McCarty, T. R., Hathorn, K. E., Redd, W. D., Rodriguez, N. J., Zhou, J. C., Bazarbashi, A. N., ... & Chan, W. W. (2020). How do presenting symptoms and outcomes differ by race/ethnicity among hospitalized patients with COVID-19 infection? Experience in Massachusetts. Clinical Infectious Diseases.
40. Li, A. Y., Hannah, T. C., Durbin, J. R., Dreher, N., McAuley, F. M., Marayati, N. F., ... & Choudhri, T. F. (2020). Multivariate analysis of black race and environmental temperature on COVID-19 in the US. The American journal of the medical sciences, 360(4), 348-356.
41. Gold, J. A., Rossen, L. M., Ahmad, F. B., Sutton, P., Li, Z., Salvatore, P. P., ... & Jackson, B. R. (2020). Race, ethnicity, and age trends in persons who died from COVID-19—United States, May–August 2020. Morbidity and Mortality Weekly Report, 69(42), 1517.
42. Jun, T., Nirenberg, S., Kovatch, P., & Huang, K. L. (2020). Mortality and risk factors among US Black, Hispanic, and White patients with COVID-19. medRxiv.

© 2021 Magesh S et al. *JAMA Network Open.*

43. Valenzuela, R. G., Michelen, Y., Bracey, A., Cruz, P., Fombonne, B., Fries, B. C., ... & Singer, A. J. (2020). Outcomes in Hispanics With COVID?19 Are Similar to Those of Caucasian Patients in Suburban New York. Academic Emergency Medicine, 27(12), 1260-1269.
44. Clouston, S. A., Natale, G., & Link, B. G. (2021). Socioeconomic inequalities in the spread of coronavirus-19 in the United States: a examination of the emergence of social inequalities. Social Science & Medicine, 268, 113554.
45. Wang, Z., Zheutlin, A., Kao, Y. H., Ayers, K., Gross, S., Kovatch, P., ... & Li, L. (2020). Hospitalised COVID-19 patients of the Mount Sinai Health System: a retrospective observational study using the electronic medical records. BMJ open, 10(10), e040441.
46. Maeda, T., Obata, R., Rizk, D., & Kuno, T. (2020). THE ASSOCIATION OF RACE, ETHNICITY, AND OUTCOMES OF PATIENTS WITH COVID-19 IN NEW YORK CITY. Chest, 158(4), A1037.
47. Selden, T. M., & Berdahl, T. A. (2020). COVID-19 And Racial/Ethnic Disparities In Health Risk, Employment, And Household Composition: Study examines potential explanations for racial-ethnic disparities in COVID-19 hospitalizations and mortality. Health Affairs, 39(9), 1624-1632.
48. Anyane?Yeboa, A., Sato, T., & Sakuraba, A. (2020). Racial disparities in COVID?19 deaths reveal harsh truths about structural inequality in America. Journal of internal medicine, 288(4), 479-480.
49. Baggett, T. P., Keyes, H., Sporn, N., & Gaeta, J. M. (2020). Prevalence of SARS-CoV-2 infection in residents of a large homeless shelter in Boston. Jama, 323(21), 2191-2192.
50. Adhikari, S., Pantaleo, N. P., Feldman, J. M., Ogedegbe, O., Thorpe, L., & Troxel, A. B. (2020). Assessment of community-level disparities in coronavirus disease 2019 (COVID-19) infections and deaths in large US metropolitan areas. JAMA network open, 3(7), e2016938-e2016938.
51. Fan, V. S., Dominitz, J. A., Eastment, M. C., Locke, E., Green, P., Berry, K., ... & Ioannou, G. N. (2020). Risk Factors for testing positive for SARS-CoV-2 in a national US healthcare system. Clinical Infectious Diseases.
52. Cordes, J., & Castro, M. C. (2020). Spatial analysis of COVID-19 clusters and contextual factors in New York City. Spatial and Spatio-temporal Epidemiology, 34, 100355.
53. Lin, C., Arevalo, Y. A., Nanavati, H. D., & Lin, D. M. (2020). Racial differences and an increased systemic inflammatory response are seen in patients with COVID-19 and ischemic stroke. Brain, behavior, & immunity-health, 8, 100137.
54. McPadden, J., Warner, F., Young, H. P., Hurley, N. C., Pulk, R. A., Singh, A., ... & Schulz, W. L. (2021). Clinical characteristics and outcomes for 7,995 patients with SARS-CoV-2 infection. PloS one, 16(3), e0243291.
55. Mendy, A., Apewokin, S., Wells, A. A., & Morrow, A. L. (2020). Factors associated with hospitalization and disease severity in a racially and ethnically diverse population of COVID-19 patients. MedRxiv.

© 2021 Magesh S et al. *JAMA Network Open.*

56. Parcha, V., Malla, G., Suri, S. S., Kalra, R., Heindl, B., Berra, L., ... & Arora, P. (2020). Geographic Variation in Racial Disparities in Health and Coronavirus Disease-2019 (COVID-19) Mortality. Mayo Clinic Proceedings: Innovations, Quality & Outcomes, 4(6), 703-716.
57. Rodriguez-Diaz, C. E., Guilamo-Ramos, V., Mena, L., Hall, E., Honermann, B., Crowley, J. S., ... & Millett, G. A. (2020). Risk for COVID-19 infection and death among Latinos in the United States: examining heterogeneity in transmission dynamics. Annals of epidemiology, 52, 46-53.
58. Amram, O., Amiri, S., Lutz, R. B., Rajan, B., & Monsivais, P. (2020). Development of a vulnerability index for diagnosis with the novel coronavirus, COVID-19, in Washington State, USA. Health & place, 64, 102377. https://doi.org/10.1016/j.healthplace.2020.102377
59. Wadhera, R. K., Wadhera, P., Gaba, P., Figueroa, J. F., Maddox, K. E. J., Yeh, R. W., & Shen, C. (2020). Variation in COVID-19 hospitalizations and deaths across New York City boroughs. Jama, 323(21), 2192-2195.
60. Blitz, M. J., Rochelson, B., Prasannan, L., Shan, W., Chervenak, F. A., Nimaroff, M., & Bornstein, E. (2020). Race/ethnicity and spatiotemporal trends in SARS-CoV-2 prevalence on obstetrical units in New York. American Journal of Obstetrics & Gynecology MFM, 100212-100212.
61. Miller, F. D., La Croix, S., Brown, T., Ramsey, L. T., & Morens, D. (2021). Unique pattern of COVID-19 infection in the State of Hawai ‘i. International Journal of Infectious Diseases, 103, 298-299.
62. Onwuzurike, C., Diouf, K., Meadows, A. R., & Nour, N. M. (2020). Racial and ethnic disparities in severity of COVID?19 disease in pregnancy in the United States. International Journal of Gynecology & Obstetrics, 151(2), 293-295.
63. McLaughlin, J. M., Khan, F., Pugh, S., Angulo, F. J., Schmidt, H. J., Isturiz, R. E., Jodar, L., & Swerdlow, D. L. (2020). County-Level Predictors of COVID-19 Cases and Deaths in the United States: What Happened, and Where Do We Go from Here?. Clinical infectious diseases : an official publication of the Infectious Diseases Society of America, ciaa1729. Advance online publication. https://doi.org/10.1093/cid/ciaa1729
64. Cahill, G., Kutac, C., & Rider, N. L. (2020). Visualizing and assessing US county-level COVID19 vulnerability. American journal of infection control.
65. Cummings, M. J., Baldwin, M. R., Abrams, D., Jacobson, S. D., Meyer, B. J., Balough, E. M., Aaron, J. G., Claassen, J., Rabbani, L. E., Hastie, J., Hochman, B. R., Salazar-Schicchi, J., Yip, N. H., Brodie, D., & O'Donnell, M. R. (2020). Epidemiology, clinical course, and outcomes of critically ill adults with COVID-19 in New York City: a prospective cohort study. medRxiv : the preprint server for health sciences, 2020.04.15.20067157. https://doi.org/10.1101/2020.04.15.20067157

© 2021 Magesh S et al. *JAMA Network Open.*

eMethods 3. Description of Statistical Methods Used in Analyses

All calculations for prevalence, relative risk ratio (RR), odds ratio (OR), and adjustment analysis were conducted using the “meta,” “metafor,” and “multicon” R packages.

The studies were separated into cohort and cross-sectional studies for all data analysis. Cohort studies include a population that is defined prior to the pandemic, while cross-sectional studies only include patients with COVID-19 at a given place/time.

A random-effects model was used to calculate all measures in this study. Random effects models were used to assess summary proportions and account for study heterogeneity, as studies included in the meta-analysis contained diverse patient populations (eFigures 5-7).

Random-effects models do not condition on the true outcomes, but instead the studies in the meta-analysis are assumed to be a random sample of the large population of studies. This is ideal for the purposes of this study, as our study population is a hypothetical population of an infinitely possible subset of study populations that may have been sampled or will be sampled in the future. In a random-effects model, the true outcomes in the studied population are assumed to be normally distributed, with μ representing the average true outcome, and $\boldsymbol{\tau}$ representing the variance of the true outcomes: $\theta \sim N(\mu, \tau^2)$. The random-effects model may also be represented as a linear combination of the average true outcome and uniformly distributed variables: $y_i = \mu + \mu_i + \varepsilon_i$, where $\mu_i \sim N(0, \tau^2)$and $\varepsilon_i \sim N(0, v_i)$ ($v_i$is the sampling variance associated with the observed outcomes).

Logit transformations were applied to all proportional data, and the Cochran’s Q test and the I^2 index were used to quantify study heterogeneity (eTable 6). Meta-regression analysis was conducted to assess correlations between study effect size and socioeconomic variables. These models were used to further examine the correlations between race/ethnicity and COVID-19 outcomes. Publication bias was assessed using the Egger’s test for publication bias. Leave-one-sensitivity analysis was conducted to examine the impact of dominating studies or outliers on results.

The relative risk ratio (RR) (with 95% confidence interval) and the odds ratio (OR) (with 95% confidence interval) describe the risk (or odds) of COVID-19 severity in different racial and ethnic groups relative to Whites. RR/OR measures were adjusted by fitting a mixed-effects model with Restricted Maximum Likelihood (REML) estimation. 8 different models were used to test for the effect of confounding variables on risk outcomes: Sex-Adjusted (Figure 1, Figure 2); Age-Adjusted (eTable 3, eTable 4); Sex & Age-adjusted (Figure 1, Figure 2); ADI-adjusted (eTable 3, eTable 4); Income-adjusted (eTable 3, eTable 4); Clinical Care-adjusted (eTable 3, eTable 4); Urban Oppurtunity Index (UOI)-adjusted (eTable 3, eTable 4); Comorbidities-

© 2021 Magesh S et al. *JAMA Network Open.*

adjusted (eTable 3, eTable 4). Studies in the unadjusted model that did not include information for one of these variables were excluded from the adjustment analysis of that particular variable. Methods to estimate missing data, such as multiple imputation, were not used as the studies were conducted separately (not a randomized trial) and the number of known outcomes would not be sufficient for accurate imputation. No more than two individual measures were adjusted at once in order to minimize the effects of overfitting (see the composite measures mentioned below).

Additionally, fitting was only calculated if the predictor variable(s) had at least 2 more outcomes than the variables being adjusted for. The mixed-effects models were fitted to the median value(s) of the variable(s) being adjusted for in order to reduce the effects of outliers. We calculated a combined measure for both Comorbidities and Clinical Care using a unit-weighted composite function, as several variables were required to appropriately adjust for these factors. The Comorbidity measure was composed using the following comorbidities that were available in the study group: ever smoker, BMI, cardiovascular disease, hypertension, COPD, diabetes, and cancer. The following variables were used to compose an estimate for the quality of Clinical care: Percent of the population under 65 that are uninsured, ratio of the population to primary care physicians, and the rate of hospital stays for ambulatory-care sensitive conditions per 100,000 Medicare enrollees (preventable hospital stays). In order to test for the similarity of variables used for the combined measures, only composed variables with a Chronbach's alpha score > 0.7 were used for adjustment (eTable 3, eTable 4). The clinical-care measure for Hispanic/Latino COVID-19 positive RR/OR was the only unit-weighted composite variable which yielded an alpha score < 0.7. Thus, RR/OR adjustment was not implemented for this cohort.

© 2021 Magesh S et al. *JAMA Network Open.*
